# Obesity-induced NLRP3 inflammasome activation in nucleus pulposus cells accelerates intervertebral disk degeneration

**DOI:** 10.1186/s13018-025-06382-y

**Published:** 2025-10-29

**Authors:** Shuai Gao, Na-Na Su, Qiang Zhao, Fang-Fang Wang, Shuan-Chi Wang, Wei Guo

**Affiliations:** 1 Department of Orthopaedics, Hebei Province Cangzhou Hospital of Integrated Traditional Chinese Medicine-Western Medicine, 31 Huanghe road, Cangzhou, 061001 People’s Republic of China; 2 Hebei Key Laboratory of Integrated Traditional and Western Medicine in Osteoarthrosis Research, Cangzhou, 061001 People’s Republic of China; 3 The Hebei Province Integrated Traditional Chinese and Western Medicine 3D Printing Technology Innovation Center, Cangzhou, 061001 People’s Republic of China

**Keywords:** Intervertebral disc degeneration, Obesity, PKR, Mitochondrial DsRNA, Pyroptosis

## Abstract

Intervertebral disc degeneration (IVDD) is a common imaging change, and it is characterized by increased production of inflammatory cytokines such as IL-1β and elevated degradation of extracellular matrix (ECM). IVDD has been indicated as the most important reason of low back pain and the leading cause of disability. IVDD is a common concomitant disease in patients with obesity; and obesity is a metabolic syndrome pathological condition that results in the concentrations of circulating fatty acid increasing and metabolic stress. Increasing circulating fatty acid could evoke a widespread inflammation response, leading to ECM degradation. Yet, how IVDD is induced by obesity, and how circulating fatty acid affects nucleus pulposus cells inflammatory response is unclear. Here we present evidence that a high fat diet (HFD) leads to IVDD and saturated fatty acid induces the activation of NLRP3 inflammasome, causing caspase-1, IL-1β production and HMGB1 release in NP cells. This involves mitochondria dsRNA (mt-dsRNA) release and double-stranded RNA-dependent protein kinase (PKR) activation. PKR deficiency inhibited NLRP3 inflammasome activation and catabolic degeneration in NP cells and rescued the phenotypes of IVDD in vivo and in vitro. Moreover, metformin prevents PKR activation and protects NP cells by attenuating mitochondria damage to NP cells. This research presents a comprehensive understanding of NLRP3 inflammasome activation mediated by mt-dsRNA-PKR axis in NP cells that underlies the development of IVDD and recommends metformin as a therapeutic drug for treating IVDD.

**Supplementary Information:**

The online version contains supplementary material available at 10.1186/s13018-025-06382-y.

## Introduction

Low back pain (LBP) is a major musculoskeletal disease with adversely affects in people of all ages and socioeconomic groups [1]. It is the most common reason for medical consultation and the leading reason of disability worldwide [1]. LBP can be caused by a variety of reasons, but intervertebral disc degeneration (IVDD) has been indicated as the most important reason [2, 3]. Intervertebral disc (IVD) is composed of nucleus pulposus (NP) located centrally, fibrous annulus in the periphery, and cartilaginous end plates that connect cranially and caudally [4]. The healthy NP tissue serves as the hydrogel-like core of the IVD, consisting mainly of NP cells and extracellular matrix (ECM) and is critical for maintaining disc mechanical function and hydration [5, 6]. In addition to mechanical stress, emerging evidence suggests that cell death pathways—such as pyroptosis, ferroptosis, and cuproptosis—which collectively contribute to IVDD progression [7]. For instance, PDK4 has been identified as a key regulator of ferroptosis in nucleus pulposus cells [8]. It is currently believed that the process of IVDD consists of gradual structural change accompanied by severe alterations in metabolic homeostasis, the formation of a pro-inflammatory microenvironment such as the increased production of IL-1β in NP cells, and elevated degradation of ECM in IVD tissue [9, 10]. Study demonstrated that NP cells are of great important for IVD function and ECM metabolism, which first exhibits degenerative changes during IVDD [11, 12].

Obesity is a chronic disease characterized by excessive body fat accumulation, and the consumption of a HFD is an important contributing factor to obesity [13]. It can affect whole body metabolism and lead to many health problems such as hypertriglyceridemia, diabetes, cardiovascular disease, arthritis, and tumors [14, 15]. The prevalence of obesity has reached almost more than two-thirds of people in the United States [16]. Increasing clinical evidence shows that obesity is closely related to the development of IVDD [17–19]. However, most of the previous studies have focused on obesity in increasing the mechanical load on the lumbar spine to cause IVDD initiation, few studies have focused on the impact of metabolic alterations due to obesity on IVD. Most obese patients have abnormally elevated blood lipid levels, and this “hypertriglyceridemia”acts as a detrimental role in all kinds of obesity complications [20, 21]. A study showed that fatty acid was a significant risk factor for IVDD even after correction for BMI and age [22]. Evidence demonstrated that increasing circulating fatty acids could evoke a widespread inflammation and stress responses, leading to ECM degradation and fibrosis [9, 23, 24]. Fatty acid treatment can lead to the activation of inflammatory signaling pathways in cells, such as TLRs and NF-κB, and promote the secretion of inflammatory factors, such as IL-1β and IL-6. Thus, the effect of fatty acid on obesity complications appears to be associated with increased inflammatory response, cell degeneration, or apoptosis. However, the influence of fatty acid on NP cells remains to be understood, and it is currently unclear whether increased circulating fatty acid has the greatest impact on IVDD in obese patients.

If the obesity induced IVDD and inflammation response systems were truly connected, it would be anticipated the involvement of pathogen sensors in NP cells inflammatory response during metabolic stress, especially during exposure to excess free fatty acids. Until now, only a few molecules are able to assume such a role with the potential ability to directly recognize pathogens and with catalytic activity that is coupled to metabolic pathways. Double-stranded RNA-dependent protein kinase (PKR) is one of such molecules. It was originally proposed as a pathogen sensor and identified as a regulator of innate immune responses which activated by recognizing long double-stranded RNAs (dsRNAs) from viruses [25]. There is increasing evidence to suggest that PKR could play a variety of roles in cellular processes besides immune response. Overactivation of PKR is found to be a hallmark of many degenerative diseases, such as Parkinson’s disease, Alzheimer’s disease, and osteoarthritis [26–29]. The activation of PKR is important to inflammasome activation [30] and PKR can respond to metabolic stress, coordinate the innate immune responses to regulate cell fate [31]. But the specific role of PKR in IVDD remains elusive.

In this research, we explored the mechanism of obesity-induced IVDD and confirmed the critical role of PKR in the pathological process of IVDD. This study confirmed that fatty acid can cause mitochondrial damage in NP cells, resulting in mt-dsRNAs efflux and increased mt-dsRNAs level in the cytoplasm. These mt-dsRNAs can induce PKR phosphorylation then leading to NLRP3 inflammasome activation and caspase-1, IL-1β production and HMGB1 release. Knock off PKR in vitro and in vivo could attenuate.

Additionally, we proved that the widely used Food and Drug Administration (FDA)-approved diabetes drug metformin attenuates mitochondrial damage to alleviate IVDD suggesting a potential therapeutic way. This study could further deepen our understanding of IVDD, and provide a new theoretical basis for its prevention and treatment.

## Materials and methods

### Mice

This study was approved by the ethics committees of Hebei Province Cangzhou Hospital of Integrated Traditional and Western Medicine (No. CZX2022090, December 6, 2022). In this study, PKR deficient (PKR-/-) mice have been established (C57BL/6JSomc-PKR^emSmoc^). Male Pkr+/+ and Pkr-/- mice were given HFD or regular diet (RD) at the age of 3 weeks and were exposed to 12 h of light /12 hours of dark cycle every day. HFD feeding at 3 weeks of age is a well-established protocol to induce diet-induced obesity in mice, as it allows for the development of obesity and its metabolic complications during young adulthood. This model effectively mimics the chronic metabolic stress of human obesity. The feeding periods of 6 and 14 months were chosen to represent early/medium-term and long-term metabolic effects on the intervertebral disc, respectively [22]. After feeding HFD or RD for 6 months and 14 months respectively, the mice were sacrificed and collected the tissues for further analysis.

## Cell preparation and stimulation

Primary Pkr+/+ (wild type) and Pkr-/- NP (PKR knock of) cells were treated with palmitic acid (PA) to evaluate changes in PKR phosphorylation levels. When the cell coverage reached 70%-80%, the cells were cultured in serum-free medium for 3 h, followed by the addition of PA. PA was dissolved in water at 65 °C to prepare a solution with a concentration of 20 mM/L. Before the experiment, 20 mM/L PA solution was diluted with DMEM containing 0.5% BSA to obtain a concentration of 0.5 mmol/L PA solution, and then treated NP cells for 6 h. PKR inhibitor Solution of 2-Aminopurinine (2-AP) for 60 min with phosphate buffer saline: glacial acetic acid (200:1) solution at 60 °C. The application concentration of 2-AP, MSU and ATP are 0.5mmol/L, 150 µg/mL, 5mmol/L respectively. The Cell lysates and precipitated supernatants samples were collected 4–6 h after treatment and analysed by Western blot or ELISA.

## Metformin application

Metformin was dissolved in drinking water at an initial dose of 205 mg/kg body weight, once a day. The dosage of metformin is converted from the human equivalent dose (1000 mg per day). This dose is a standard conversion from the typical human clinical dose using body surface area normalization guidelines and is widely used in mouse studies to achieve therapeutic relevance [32, 33]. We measured the water intake and body weight of mice once a week, and adjusted the concentration of metformin in drinking water weekly according to the changes of water intake and body weight of mice. Mice in the control group and IVDD model group were killed 12 weeks after the operation.

## Immunofluorescence

Cultured mouse NP cells were fixed on covered glass with 4% formalin (20 min at room temperature), followed by treatment with 0.1% Triton X-100 and 0.2% Tween-20 (40 min at room temperature) and blocked with 5% non-fat milk. The samples were incubated with primary antibody (anti-PKR, anti-pPKR, anti-HMGB1, anti-GSDMD-N, anti-ASC). After incubation, the cells were washed three times with PBS solution for 3 min each time, and the fluorescent secondary antibody was added and incubated for 1 h before nuclei were restained with DAPI. The images were captured with a fluorescence microscope (Leica).

## Immunohistochemistry

The mice were killed by CO_2_ anesthesia. The tissue samples were fixed with 4% paraformaldehyde for 48 h, followed by 10% EDTA decalcification and paraffin embedding. Tissue sections were used for safranin-o/fast green staining, immunohistochemistry and immunofluorescence staining. The primary antibodies include IL-1β, caspase-1, GSDMD-N, collagen-II, aggrecan, MMP-13 and ADAMTS-5 antibodies. Histological images were analyzed under a BX53 microscope (Olympus).

### Western blotting

Cells were lysed with a buffer containing 0.25 M Tris-HCl, 20% glycerol, 4% sodium dodecyl sulfate (SDS), and 10% mercaptoethanol (pH 6.8), and protease and phosphatase inhibitors were added. Equal amounts of total protein (10 µg) were separated by 10–12% SDS-polyacrylamide gel and electrotransferred to polyvinylidene fluoride membrane. 5% non-fat milk was added to tris buffered saline containing 0.1% Tween-20 (TBST), blocked at room temperature for 1 h, and incubated with primary antibody in TBST containing 5% non-fat milk at 4 °C overnight. Secondary antibody was added at room temperature for 1 h and western blotting was performed using an enhanced chemiluminescence system.

## ELISA

The concentrations of mouse IL-1β, IL-6, TNF-α, IL-18, and HMGB1 in the cell culture supernatant were quantified using commercially available ELISA kits according to the manufacturers’ instructions. The kits used were: Mouse IL-1β ELISA Kit (R&D Systems, Catalog # MLB00C), Mouse IL-6 ELISA Kit (R&D Systems, Catalog # M6000B), Mouse TNF-α ELISA Kit (R&D Systems, Catalog # MTA00B), Mouse IL-18 ELISA Kit (MBL, Catalog # 7625), and Mouse HMGB1 ELISA Kit (Tecan, Catalog # ST51011).

## Quantitative reverse-transcription PCR (RT-qPCR)

After chloroform extraction and precipitation, the DNA in the sample was removed by DNase I treatment, followed by reverse transcription of purified RNA using RevertAid reverse transcriptase. The reverse transcription primers containing CMV promoter sequences were designed to target strand specific genes. AriaMx Real-time PCR system and QuantStudio Real-time PCR system were used for RT-qPCR. The primers used in this study are shown in supplementary Tables S1 and S2.

### Cell transfection

Lipofectamine 3000 (Invitrogen) was used to transfect the plasmids or siRNA respectively with third-generation mouse NP cells as recommended by the manufacturer and described previously [34]. Cells were collected 48 h after transfection.

### Mitochondrial membrane potential

Mitochondrial membrane potential was stained with JC-1 dye. After washing with PBS once, they were incubated in preheated medium containing 5 µg/mL JC-1 dye in a 5%CO2 incubator at 37 °C for 30 min. The cells were then washed 3 times with PBS and imaged with Leica fluorescence microscope.

### PKR CLIP and RNA sequencing

We prepared PKR antibody conjugated beads by incubating protein A beads with PKR antibody in CLIP lysis buffer at 4 °C for 3 h. The NP tissue sample was lysed on ice for 10 min and then sonicated. Centrifugation was used to separate debris. PKR antibody conjugated beads were added into the lysate and incubated at 4 °C for 3 h. The beads were washed with CLIP washing buffer 4 times and incubated in the elution buffer at 25 °C for 3 h to elute the PKR-RNA complex from the beads. The eluate was treated with 20 mg/mL proteinase K for overnight at 65 °C. RNA was purified using acidphenol: Chloroform, pH 4.5. cDNA of target size were screened using AMPure XP beads for PCR amplification and again purified PCR products were used in AMPure XP beads to obtain a library. Using Agilent 2100 Bioanalyzer (Agilent Technologies Inc., California, USA), Agilent High Sensitivity DNA Kit (Agilent Technologies Inc., California, USA, 5067 − 4626) for library quality detection. Total library concentration was detected by Pico green (Quantifluor-ST fluorometer, Promega, Madison, Wisconsin, USA, E6090; Quant - iT PicoGreen dsDNA Assay Kit, Invitrogen, California, USA, P7589). Libraries were sequenced on an Illumina HiSeq-2000 with 150-bp paired-end reads, v3 chemistry.

### Statistical analysis

Each experiment was repeated at least three times, and cells in each experiment were obtained from a single isolation process. Continuous data were expressed as mean ± standard deviation. The student t test was used for comparison between the two groups. All comparisons were predefined and hypothesis-driven. Given the limited number of independent tests per experiment, multiple comparison corrections were not applied to avoid overcorrection and loss of statistical power. Statistical analysis was performed using Prism 7.0 version (GraphPad Software, La Jolla, CA) or SPSS 22.0 version (SPSS Inc., Chicago, IL). Significance levels were set at *P < 0.05, **P < 0.01, and ***P < 0.001.

## Results

### PKR activation in high-fat diet induced IVDD

To explore whether hypertriglyceridemia could accelerate the IVDD, we first observed the IVDD from the wild-type (WT) mice fed with either RD or HFD for 6 or 14 months. After 6 months of HFD, the body weight of mice increased significantly (*P* = 1.52 × 10^− 5^). The mean body weight of HFD mice was more than 40% higher than that of RD mice, indicating that the target level of obesity was achieved. The levels of fasting blood glucose (*P* = 4.04 × 10^− 9^), non-esterified fatty acids (*P* = 1.66 × 10^− 8^), and triglycerides (*P* = 1.25 × 10^− 15^) in mice with HFD were higher than those in RD. However, the increase in total cholesterol was not significant (Table 1). Similarly, these obesity-related traits, except total cholesterol, remained substantially elevated at 14 months in the HFD group (Table 1).

**Table 1:** Data for mice fed with regular diet (RD) compared with high fat diet (HFD)

	6 months				14 months		
	RD (*n* = 10)	HFD (*n* = 10)	*P* value		RD (*n* = 10)	HFD (*n* = 10)	*P* value
Weight (g)	30.97 ± 4.21	43.48 ± 3.81	1.52 × 10^− 5^		43.28 ± 4.26	59.98 ± 3.89	3.44 × 10^− 8^
Fast blood glucose (mmol/L)	5.90 ± 0.65	9.60 ± 0.90	4.04 × 10^− 9^		6.78 ± 0.72	11.13 ± 1.29	2.61 × 10^− 8^
Non-esterified fatty acid (mmol/L)	230.62 ± 19.20	300.81 ± 12.87	1.66 × 10^− 8^		244.17 ± 14.75	312.70 ± 16.03	9.63 × 10^− 9^
Triglyceride (mmol/L)	0.32 ± 0.06	0.94 ± 0.05	1.25 × 10^− 15^		0.34 ± 0.05	1.03 ± 0.05	1.28 × 10^− 17^
Total cholesterol (mmol/L)	1.36 ± 0.07	1.49 ± 0.22	0.09		1.38 ± 0.06	1.51 ± 0.21	0.06

**Fig. 1:**
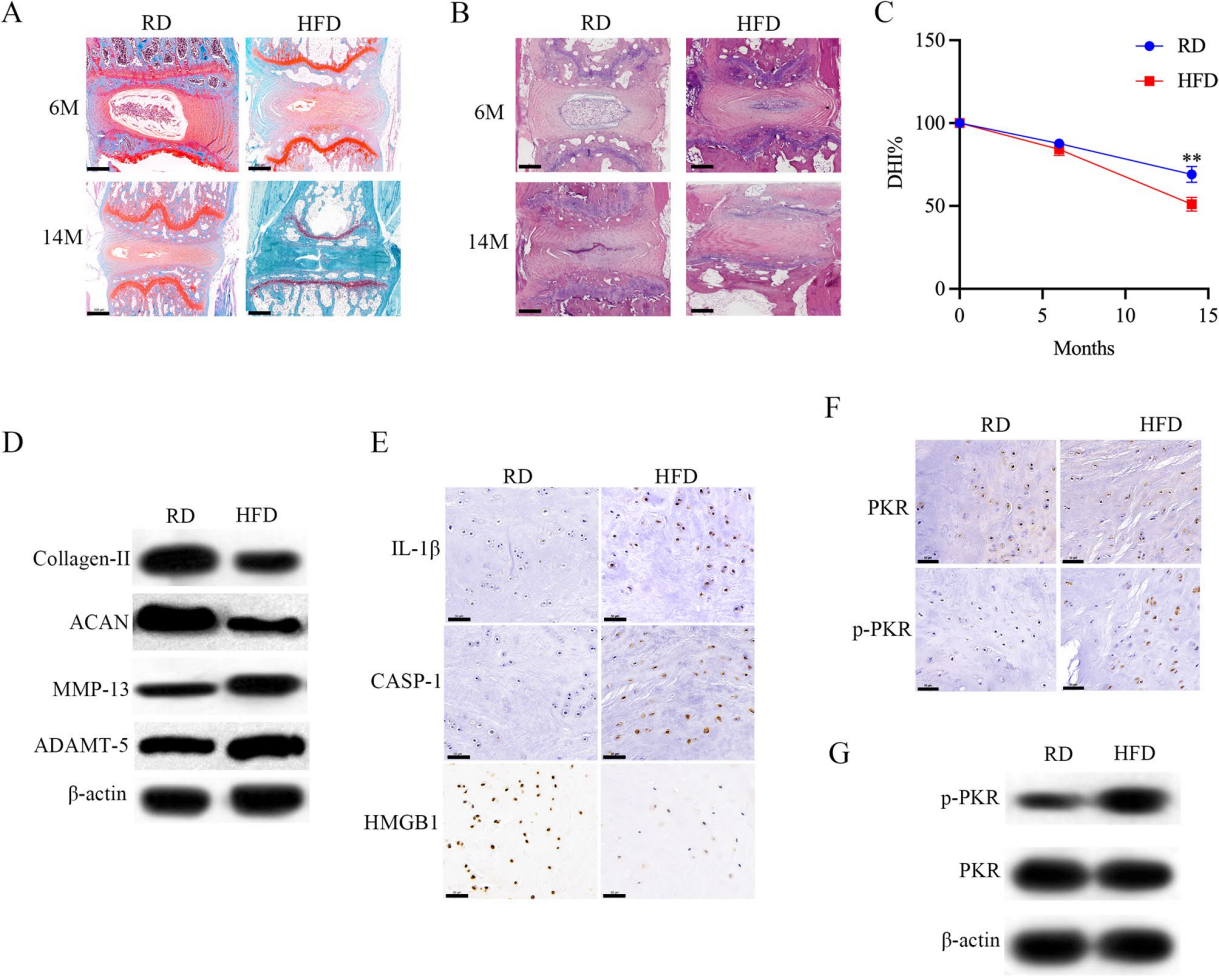
PKR activation in metabolic stress induced IVD degeneration. **A**, **B** Safranin-O/fast green and Hematoxylin and Eosin staining of IVD tissue from the wild-type (WT) mice treated with either RD or HFD for 6 or 14 months; scale bar = 500 μm, *n* = 3, **C** changes in disc height index (DHI) of the indicated groups. The DHI was measured at 0, 6, and 14 months. A significant decrease of the DHI% was observed in 14 months after HFD fed (*P* < 0.01), **D** Western blot analysis of collagen-II, ACAN, MMP-13, and ADAMT-5 protein levels of NP tissue from WT mice treated with either RD or HFD for 14 months, **E** immunohistochemistry staining of NP tissue from WT mice treated with either RD or HFD for 14 months; scale bar = 50 μm, *n* = 3, **F**, **G** PKR activity was examined in NP tissue of WT mice kept either on a RD or HFD for 14 months; scale bar = 50 μm, *n* = 3

Safranin-O/fast green staining and Hematoxylin and Eosin staining of IVD tissue showed more obvious IVD degeneration morphology change in HFD treated mice (Fig. 1A, B). Disc height was measured at the mid-sagittal plane of IVD tissues from RD- and HFD-fed mice (*n* = 3 per group) using ImageJ software. DHI was calculated as the ratio of disc height to adjacent vertebral body height. HFD-fed mice exhibited a significant reduction in DHI compared to RD controls (*P* = 0.008; Fig. 1C). Subsequently, western blotting results (Fig. 1D) showed that extracellular matrix components collagen-II and aggrecan were down-regulated in HFD treated mice, whereas the matrix degrading enzyme MMP-13 and ADAMT-5 were increased markedly. Moreover, IL-1β, caspase-1 expression and high mobility basal box 1 (HMGB1) release from the NP cell nucleus were also up-regulated in HFD treated mice (Fig. 1E). Early observations suggested that HMGB1 release from the nucleus is highly associated with pyroptosis [35]. In Fig. 1F, G, we also observed an increased phosphorylated PKR protein level in NP tissue from HFD treated mice. Since these mice were kept in a specific pathogen-free environment, PKR activation probably associated with signals related to HFD. These results reminded us that HFD induced IVDD is probably connected with PKR activation, and this activation is associated with the expression of inflammasome related cytokines.

### Role of PKR in palmitic acid mediates NP cells pyroptosis

**Fig. 2:**
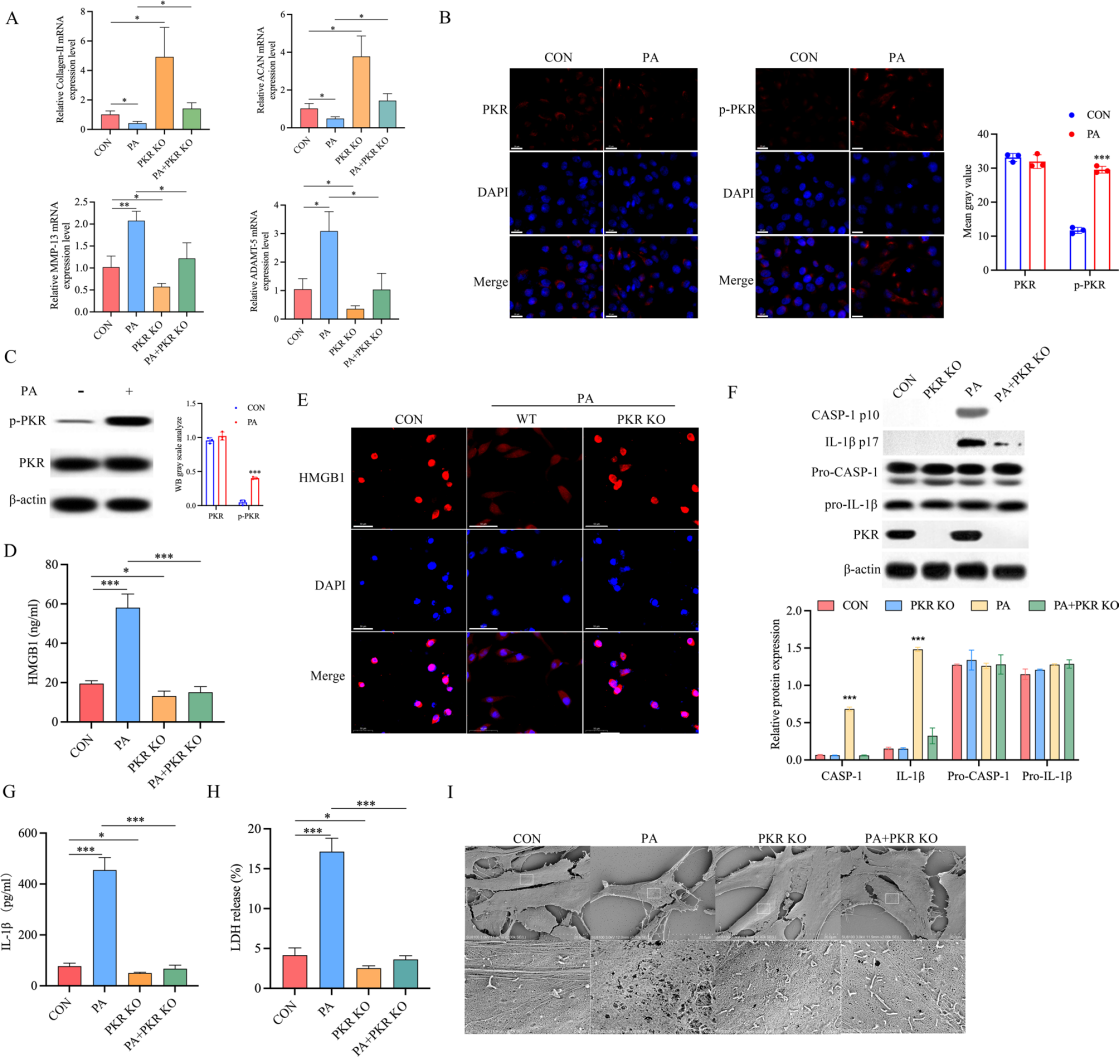
The Role of PKR in palmitic acid mediates NP cells pyroptosis. NP cells from PKR+/+ or PKR-/- (PKR KO) mice NP tissue was treated with palmitic acid or not. **A** qRT–PCR analysis of collagen-II, ACAN, MMP-13, and ADAMT-5 mRNA expression in each group. *p < 0.05, **p < 0.01 by Student’s t test, *n* = 3, **B**, **C** PKR (red) activity was examined in NP cells treated with palmitic acid by immunofluorescence staining and western blot; Nucleus (DAPI), scale bar = 20 μm, *n* = 3, **D** HMGB1 level in the supernatant was determined by ELISA in each group; *p < 0.05, ***p < 0.001 by Student’s t test, *n* = 3, **E** intracellular HMGB1, visualized with red immunofluorescence staining, is predominately localized in the nucleus in NP cells. Following by exposure to palmitic acid, nuclear HMGB1 is released from the nucleus to the cytoplasm and knock off PKR significantly inhibited HMGB1 release. HMGB1 (red), DAPI (blue), scale bar = 50 μm; **F** Caspase-1 activation and IL-1β cleavage were assessed by Western blot, *n* = 3. PKR+/+ or PKR-/- NP cells were stimulated as indicated. **G**, **H**) PKR+/+ or PKR-/- NP cells were treated with palmitic acid as indicated; Supernatant was detected by ELISA. Cytotoxicity was determined by LDH assay, *p < 0.05, ***p < 0.001 by Student’s t test, *n* = 3, **I** the scanning electron microscopy showed that membrane pore-forming was increased by palmitic acid and PKR knock off decreased the membrane pore-forming in NP cells induced by palmitic acid; scale bar = 20 μm (below: magnification of the tetragonum; scale bar = 2 μm). PA: palmitic acid

PA is one of the most abundant saturated fatty acids in plasma and is significantly elevated following a HFD [36]. Given that IVDD is likely induced by HFD which increases circulating lipid levels, we treated NP cells with palmitic acid (PA) to explore specific mechanisms in vitro. RT-PCR results demonstrated that PA treatment induced a degenerative phenotype in NP cells: relative expressions of anabolism genes (aggrecan, collagen-II) were significantly down-regulated while the catabolism genes MMP-13 and ADAMT-5 were significantly up-regulated, and PKR knock off significantly attenuate this degenerative phenotype induced by PA (Fig. 2A). As shown in Fig. 2B, C, we observed increased PKR phosphorylation level in NP cells exposed to PA. We also observed that in the PA induced degenerative NP cells, HMGB1 release from the nucleus to the cytoplasm was significantly increased and knock off PKR significantly inhibited HMGB1 release (Fig. 2D, E). Early observations suggested that HMGB1 release from the nucleus is highly associated with cell pyroptosis [35]. To address the effect of PKR in NP cells pyroptosis, we next inspect the caspase-1 activation and IL-1β cleavage in NP cells. We treated primary isolated NP cells from PKR−/− or PKR+/+ mice with PA. The results showed that PKR knock off significantly inhibits the PA induced caspase-1 activation and IL-1β cleavage in NP cells (Fig. 2F). Similarly, treating wild type NP cells with ATP or MSU, two stimulants that promote pyroptosis, resulted in prominent induction of caspase-1 activation and IL-1β cleavage in NP cells compared with PKR knock off NP cell (Supplementary Fig. 1A). We also observed that the release of IL-1β promoted by exposing to PA or MSU was markedly attenuated in PKR Knock NP cells compared with wild type cells (Supplementary Fig. 1B). Moreover, PKR knock off markedly attenuates LDH and IL-1β release in PA treated NP cell (Fig. 2G, H). And PKR knock off also decreased the membrane pore-forming in NP cells induced by PA treatment (Fig. 2I). These results indicated that NP cell pyroptosis occurs after PA treated, and this pyroptosis of NP cell is mediated by PKR.

### PKR controls NLRP3 inflammasome activation during NP cell pyroptosis

**Fig. 3:**
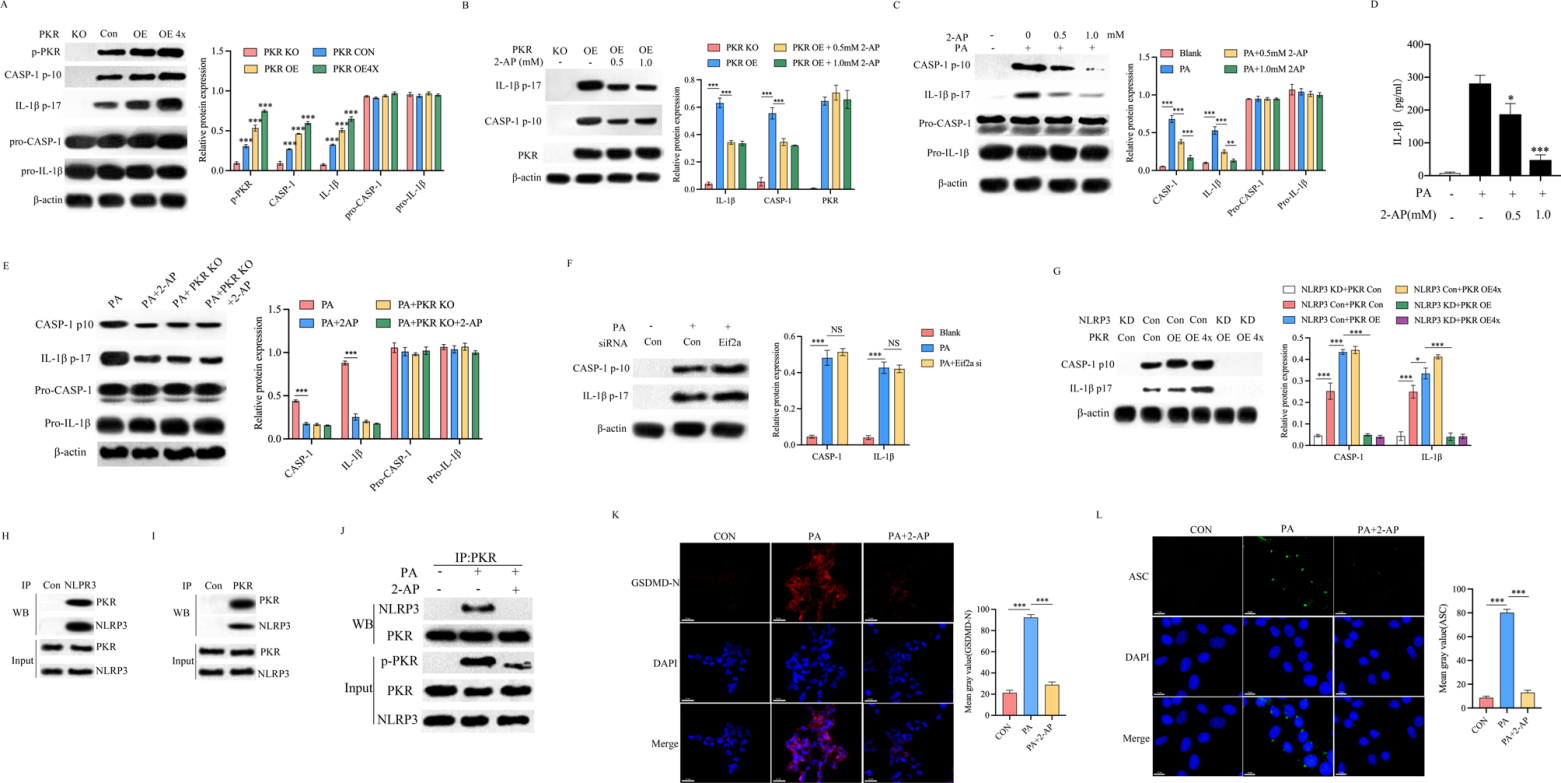
PKR controls NLRP3 inflammasome activation in response to metabolic stress. **A**, **B**) NP cells were transfected as indicated. Caspase-1 activation and IL-1β cleavage were assessed by Western-blot, *n* = 3, **C**, **D** PKR+/+ NP cells were treated with palmitic acid and 2-AP as indicated. Caspase-1 activation and IL-1β cleavage were assessed by Western blot and ELISA*p < 0.05, ***p < 0.001 by Student’s t test, *n* = 3, **E** PKR+/+ or PKR-/- NP cells were treated with palmitic acid and 2-AP as indicated, **F**, **G** PKR+/+ NP cells were transfected as indicated. Caspase-1 activation and IL-1β cleavage were assessed by Western blot, *n* = 3, **H**, **I** immunoprecipitation and Western-blot analysis of the physical interaction of PKR and NLRP3 in PKR+/+ NP cells using recombinant proteins or **J**, **K** PKR+/+ NP cells treated with palmitic acid or 2-AP as indicated, *n* = 3. **L**, **M** The immunofluorescence staining for detection of CSDMD-N (red) and ASC (green) in the PKR+/+ NP cells treated as indicated; Nucleus (DAPI), scale bar = 20 μm (L), scale bale = 10 μm (M). PA: palmitic acid

As one of the most widely researched multiprotein complex, NLRP3 inflammasome is a key regulator of IL-1β release and caspase-1 activation [37]. Recent studies have shown that activation of the NLRP3 inflammasome is associated with IVD inflammation, pyroptosis, extracellular matrix degradation and death of IVD cells [38–40]. However, the factors that lead to the activation of NLRP3 inflammasomes during IVDD are unclear. In this research, we investigated the effect of PKR on pyroptosis and NLRP3 inflammasome activation by activating or inhibiting PKR activity in vitro (Fig. 3). The results demonstrated that PKR overexpression prominently facilitates caspase-1 activation and IL-1β cleavage (Fig. 3A); And these phenomena could be attenuated by inhibiting PKR activity pharmacologically with 2-AP (Fig. 3B). Suppress PKR activity by 2-AP could also attenuate PA induced caspase-1 activation and IL-1β cleavage and release (Fig. 3C, D). Moreover, 2-AP failed to further suppress caspase-1 activation and IL-1 β cleavage in PKR knock off NP cells (Fig. 3E). Accordingly, These findings suggest a critical role for PKR in activation of the NLRP3 inflammasome during pyroptosis of NP cell. As one of the main substrates of PKR, eukaryotic initiation factor 2a (Eif2a) could regulate protein synthesis in general [41]. It would seem to be logical that the effect of PKR on inflammasome activity is mediated by Eif2a. However, we observed that Eif2a deficiency did not alter caspase-1 activation and IL-1β cleavage after PA treatment (Fig. 3F). Moreover, PKR overexpression does not directly activate caspase-1 and IL-1β when NLRP3 is absent (Fig. 3G). Therefore, we infered that PKR facilitates inflammasome activation through direct interaction with NLRP3. We prepared mouse NP cells lysates for immunoprecipitation using anti-PKR antibody. The results indicated that PKR pulled down NLRP3, and reciprocal immunoprecipitation of NLRP3 pulled down PKR (Fig. 3H, I). Moreover, exposure of NP cells to PA prominently facilitated NLRP3 and PKR complexes formation (Fig. 3J), and pharmacological inhibition of PKR activation by 2-AP markedly restrained the NLRP3-PKR complexes formation (Fig. 3J). Moreover, the proportion of GSDND-N and ASC specks was increased in NP cells after PA treatment, indicating that the inflammasome is activated in response to PA; and inhibit PKR activation by 2-AP could eliminate the effect of PA on NP cells (Fig. 3K, L). Together with evidence that GSDMD is the key executor of NLRP3 inflammasome induced pyroptosis [42], we considered that the PKR is critical for the activation of NLRP3 inflammasome during NP cells pyroptosis.

### mt-dsRNAs are the major class of cellular dsRNAs interacting with PKR in response to metabolic stress in NP cells

**Fig. 4:**
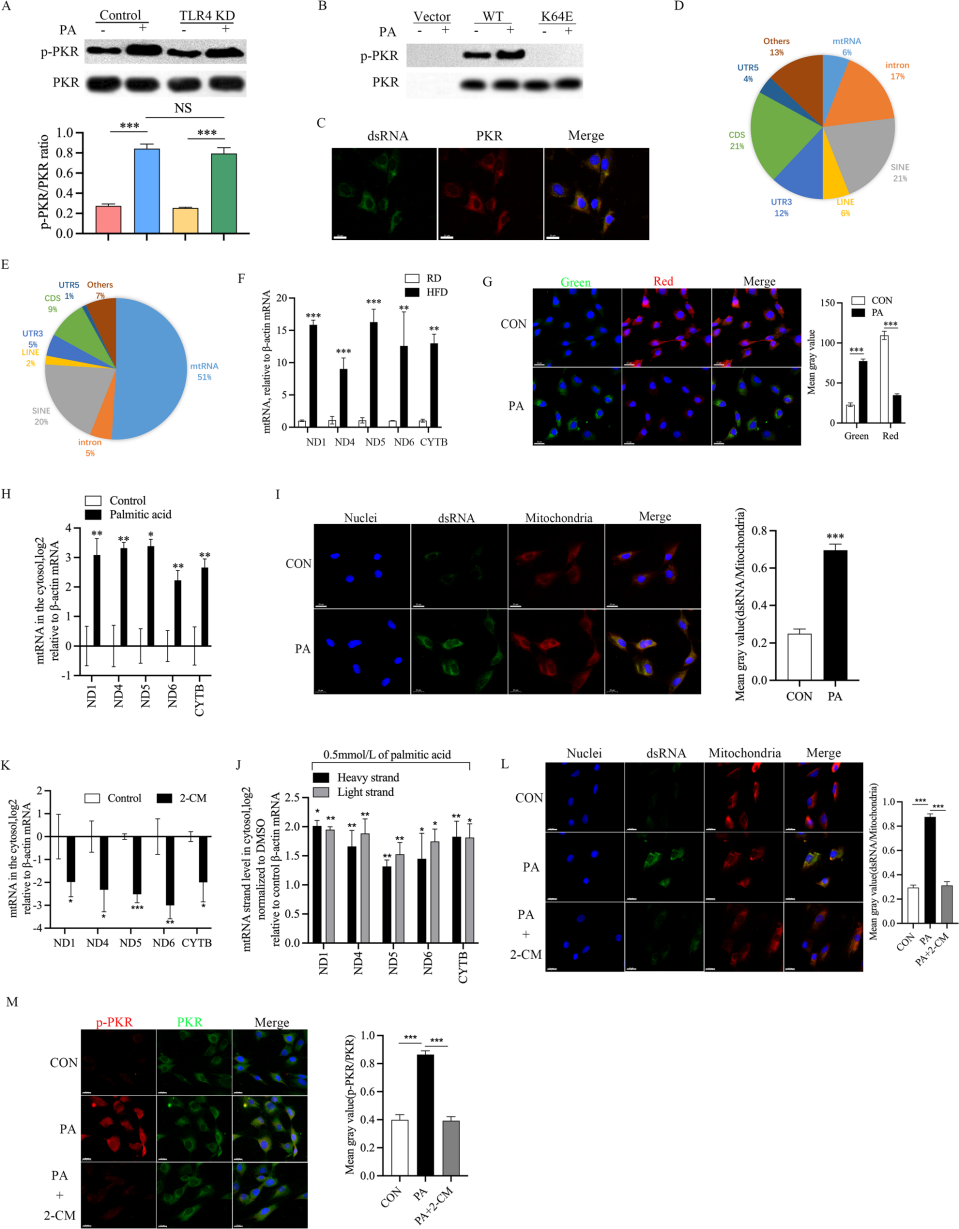
mt-dsRNAs are the major class of cellular dsRNAs interacting with PKR in response to metabolic stress in NP cells. **A** Palmitic acid induced PKR activation in TLR4 Knock down NP cells, *n* = 3, **B** palmitic acid induced PKR activation requires intact RNA-binding domain of PKR. PKR-/-NP cells were transfected with vector, wild-type (WT) or RNA-binding domain mutant (K64E) of PKR by retrovirus-mediated gene transfer, *n* = 3, **C** Co-localization of dsRNA (green) with PKR (red) in response to palmitic acid treatment was analyzed via immunofluorescence staining, Nucleus (DAPI), scale bar = 20 μm, *n* = 3 **D**, **E** the relative amount of sequencing reads from the CLIP-seq libraries by RNA classes, **F** PKR-mtRNAs interaction upon palmitic acid treatment examined by PKR CLIP-qPCR analysis, *p < 0.05, **p < 0.01, ***p < 0.001 by Student’s t test, *n* = 3, **G** the effect of palmitic acid on the mitochondrial membrane potential. Nucleus (DAPI), scale bar in = 20 μm, *n* = 3, **H** cytosolic mtRNAs expression in palmitic acid treated NP cells, *p < 0.05, **p < 0.01 by Student’s t test, *n* = 3, **I** immunofluorescence staining of dsRNAs (green) in palmitic acid treated NP cells with anti-dsRNAs (J2) antibody. Mitochondria and nuclei are stained with MitoTracker Deep Red and Hoechst (blue), respectively. scale bars = 20 μm, *n* = 3, **J** cytosolic strand-specific mtRNAs expression in palmitic acid treated NP cells, *p < 0.05, **p < 0.01 by Student’s t test, *n* = 3, **K** the effect of 2-CM treatment on mtRNAs expression was analyzed by RT-qPCR, *p < 0.05, **p < 0.01, ***p < 0.001 by Student’s t test, *n* = 3. (L) The effect of 2-CM on mt-dsRNAs (green) expression and the phosphorylation of PKR (p-PKR = red, PKR = green) in palmitic acid treated NP cells. Nucleus (DAPI), scale bar = 20 μm, *n* = 3. PA: palmitic acid

Considering TLR4 is able to sense fatty acid signal and induce an inflammatory response [43], we asked whether this pattern recognition receptor is involved in the activation of PKR induced by PA. However, the level of PA -induced PKR activation was not affected after TLR4 knockout (Fig. 4A). These results indicated that PKR activation by PA is not dependent on TLR4. Previous research demonstrated that as a sensor for cell stress, PKR undergoes autophosphorylation by recognizing endogenous dsRNAs through its RNA binding domains [44]. We next enquired that whether the RNA-binding capacity of PKR is necessary for PA induced PKR activation. We abolished the RNA binding ability of PKR by inserting a point mutation of lysine 64 residue into the RNA binding motif of PKR [45, 46]. We transfected wild-type PKR gene or RNA-binding defect (K64E) PKR gene into PKR-/- NP cells and detected PKR kinase activity after treated with PA. Wild-type PKR was activated by PA (Fig. 4B). However, PKR with RNA binding deficiency (K64E) was not activated by PA (Fig. 4B). In addition, the dsRNAs in the cytoplasm after PA treatment were co-localized with PKR, which suggests that the activation of PKR may be related to dsRNAs in the cytoplasm. To seek out the upstream activator of PKR during IVDD, we conducted formaldehyde mediated crosslinking and immunoprecipitation sequencing (CLIP-seq) analysis. Of note, the amount of mtRNAs interacting with PKR was increased in NP tissue after treated HFD (Fig. 4D-E). Next, we used qRT-PCR to detect the levels of mtRNAs in NP tissues, and the results showed that mtRNAs expression levels were significantly increased in HFD-induced degenerative NP tissues (Fig. 4F). In the cell experiments, we found that PA treatment led to mitochondrial membrane potential damage (Fig. 4G), increased mtRNA level (Fig. 4H) and dsRNA levels in the cytosol (Fig. 4I). To prove that these mtRNAs in the cytoplasm were indeed dsRNAs, we conducted strand specific reverse transcription and observed that both heavy and light transcripts were increased in the cytoplasm after PA treatment (Fig. 4J). Previous studies have found that, in addition to mt-dsRNAs, cells encode multiple types of non-coding RNAs and repeat sequences that can lead to PKR activation [47]. To verify whether mt-dsRNAs are the major contributors to PKR activation during IVDD, we used 2-CM to inhibit mitochondrial RNA polymerase activity to reduce intracellular mt-RNA production (Fig. 4K). We observed the up-regulation of mt-dsRNAs and activation of PKR in NP cells induced by PA was attenuated upon 2-CM treatment (Fig. 4L). Together, these results suggest that the damaged mitochondrial releases mt-dsRNAs into the cytoplasm and then activates PKR during IVDD.

### Metformin inhibits PKR activation and NP cell pyroptosis by reducing mt-dsRNAs expression

**Fig. 5:**
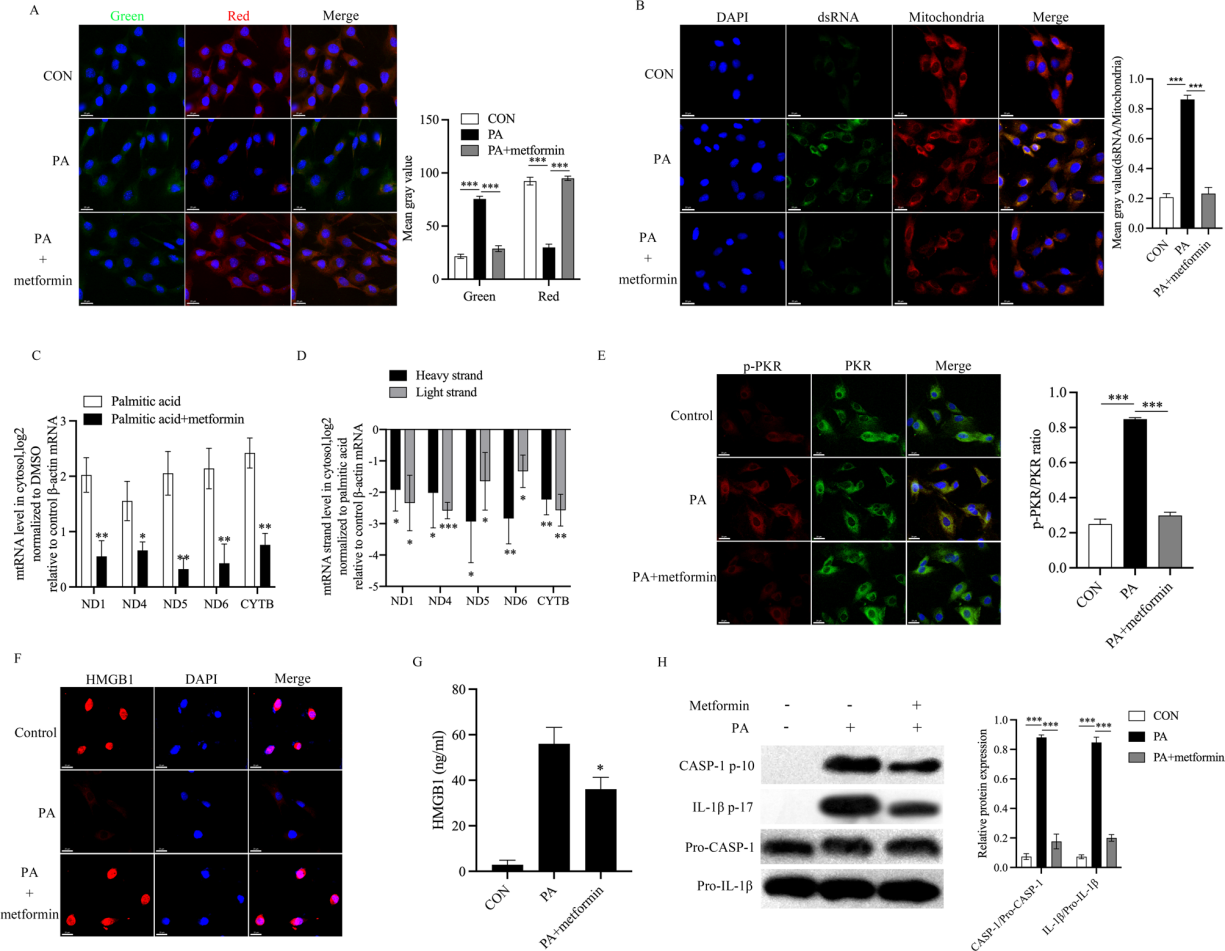
Metformin inhibits PKR activation and NP cell pyroptosis by reducing mt-dsRNAs expression. **A** The effect of metformin on mitochondrial membrane potential in palmitic acid treated NP cells, scale bar = 20 μm, *n* = 3, **B** the effect of metformin on dsRNAs (green) expression in palmitic acid treated NP cells. Mitochondria (red) and nuclei are stained with MitoTracker Deep Red and Hoechst (blue), respectively, scale bars = 20 μm, *n* = 3, **C**, **D** Tte effect of metformin on Cytosolic total or strand-specific mtRNAs expression in palmitic acid treated NP cells, *p < 0.05, **p < 0.01, ***p < 0.001 by Student’s t test, *n* = 3, **E** the effect of metformin on the phosphorylation of PKR in palmitic acid treated NP cells (p-PKR = red, PKR = green), scale bar = 20 μm, Nucleus (DAPI), *n* = 3, **F**, **G**. The effect of metformin on HMGB1 (red) release in palmitic acid treated NP cells were assessed by immunofluorescence staining and ELISA, Nucleus (DAPI), scale bar = 20 μm, *p < 0.05 by Student’s t test, *n* = 3, **H** the effect of metformin on caspase-1 activation and IL-1β cleavage in palmitic acid treated NP cells were assessed by Western blot. NP cells were stimulated as indicated, *n* = 3. PA: palmitic acid

Evidence suggests that metformin affects a variety of cellular processes, including the inflammatory response [48–50]. Interestingly, we observed that metformin was able to inhibit the damage to mitochondrial membrane potential caused by PA (Fig. 5A). We subsequently observed a decrease of mt-dsRNAs expression in PA treated NP cells after metformin application (Fig. 5B). Next, pretreated NP cells with metformin decreased the cytosolic level of mt-RNAs compared with the PA treated group (Fig. 5C, D). To confirm the protective effect of metformin on mitochondria, we used oligoA, an ATP synthase inhibitor of the mitochondrial respiratory chain complex, to treat NP cells. The results showed that oligoA treatment leads to mitochondrial membrane potential damage and mt-dsRNAs increase in the cytosol (Supplementary Fig. 2A-B). Metformin rescued mitochondrial damage from oligoA and reduced the mt-dsRNAs release in NP cells (Supplementary Fig. 2A-B). We also found that metformin also inhibits PKR activation in NP cells (Fig. 5E). In addition, the HMGB1 release, IL-1β cleavage and caspase-1 activation also reduced by metformin treatment (Fig. 5F, H). These results indicated that the metformin inhibits NP cells pyroptosis by attenuating mitochondrial damage and PKR activation.

### Metformin inhibits IVDD in the mouse IVD degeneration model

**Fig. 6:**
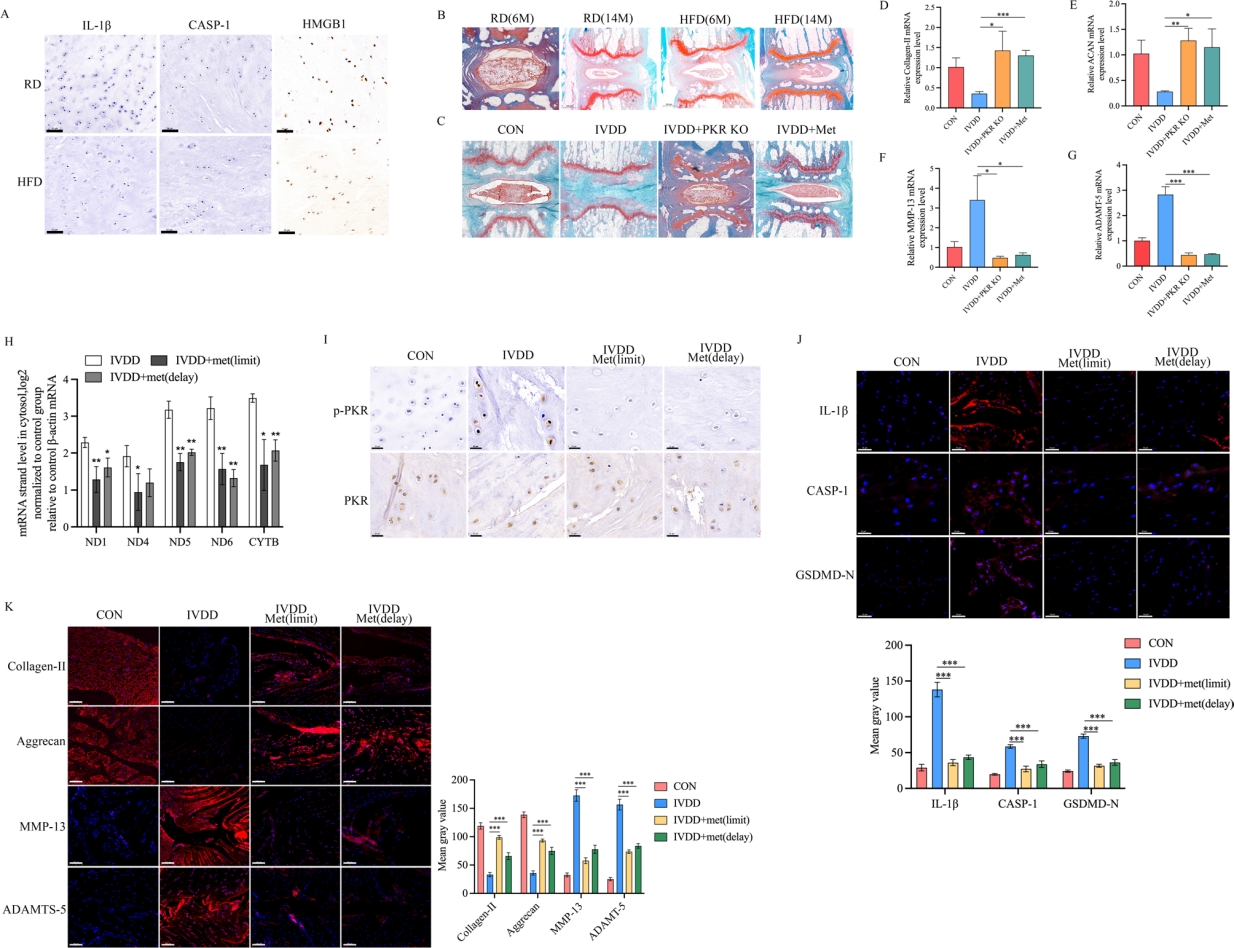
Metformin ameliorates IVD degeneration development in the mouse IVD degeneration model. **A** Immunohistochemistry staining of NP tissue from PKR+/+ and PKR-/- mice treated with either RD or HFD for 14 months; scale bar = 50 μm, *n* = 3, **B** Safranin-O/fast green staining of IVD tissue from the PKR-/- mice treated with either RD or HFD for 6 or 14 months; scale bar = 500 μm, *n* = 3, **C** IVD tissue from control, IVDD (12 weeks after surgery, PKR knock off or not) or treatment with metformin (limit) were assessed using safranin-O/fast green staining, **D**–**G** qRT–PCR analysis of collagen-II, ACAN, MMP-13, and ADAMT-5 mRNA expression in each group. *p < 0.05, **p < 0.01, ***p < 0.001 by Student’s t test, *n* = 3, **H** the expression of mtRNAs in the NP tissue of IVDD, metformin treatment (limit or delay) group normalized by the mtRNAs expression of control group, *p < 0.05, **p < 0.01 by Student’s t test, *n* = 3, **I** immunohistochemistry of p-PKR and PKR on the NP tissue of control, IVDD and metformin treatment (limit or delay) group, scale bar = 20 μm, *n* = 3, **J** immunofluorescence staining for IL-1β (red), caspase-1 (red) and GSDMD-N (red) in the IVD degeneration model mice treated with metformin (limit or delay). Nucleus (blue), scale bar = 50 μm, *n* = 3, **K** immunofluorescence staining for collagen-II (red), aggrecan (red), MMP-13 (red) and ADAMTS-5 (red) in the IVD degeneration model mice treated with metformin (limit or delay). Nucleus (blue), scale bar = 100 μm, *n* = 3

We used PKR deficient (PKR-/-) mice with either RD or HFD treated to observe the IVD morphology. Our results showed that when PKR was knocked off, the expression of IL-1β and caspase-1 and the release of HMGB1 were not significantly different between RD and HFD groups (Fig. 6A). Safranin-O/fast green staining of IVD showed that there is no significant difference in NP tissue morphology between the RD group and HFD group (Fig. 6B). These results demonstrated that HFD failed to induce pyroptosis of NP cells and IVDD when PKR is deficiency. To further validate the results of this study and examine their clinical relevance, we performed IVD needle puncture surgery to induce IVDD in wild-type and PKR-/- mice aged 12 weeks with or without metformin application (4 mg/day in drinking water, until the animals were sacrificed) beginning at 1 week before (limit or prevention) or 1 week after (delay or treatment) surgery. We performed safranin-O/fast green staining to evaluate IVDD 12 weeks after surgery. We observed that IVDD was obvious at 12 weeks after surgery, and the progression of IVDD can be attenuated by PKR knock off or application of metformin 1 week before surgery (Fig. 6C). Subsequently, qRT-PCR analysis showed that PKR knock off or metformin application markedly inhibited catabolic degeneration of NP tissues and rescued these phenotypes of IVDD. Moreover, the content of mtRNAs was significantly increased in the NP tissue from IVDD mice, which was consistent with the results of cell experiments, and the application of metformin could significantly reduce the content of mtRNAs in the NP tissue (Fig. 6H). In order to verify whether the increased mtRNAs in NP tissues result in the PKR activation in vivo, we preformed immunohistochemistry analysis to observe p-PKR staining in NP tissues. We found an increased signal for p-PKR in degeneration NP tissue compared to control, this effect was prominently attenuated by application of metformin starting either before or after surgery (Fig. 6I). We further tested the effect of metformin on activation of inflammasome and expression of inflammatory factors. The results showed that in the application of metformin either before or after needle puncture surgery, inflammasome activation was prominently reduced (Fig. 6J). Previous studies demonstrated that the ECM integrity is crucial for proper IVD function. In this study, we found metformin treatment significantly inhibits collagen-II, aggrecan losing and reduces MMP13, ADAMTS-5 expression in NP tissue (Fig. 6K). These results indicated that metformin can inhibit the occurrence of IVDD and delay the progression of IVDD in the mouse IVDD model.

## Discussion

**Fig. 7:**
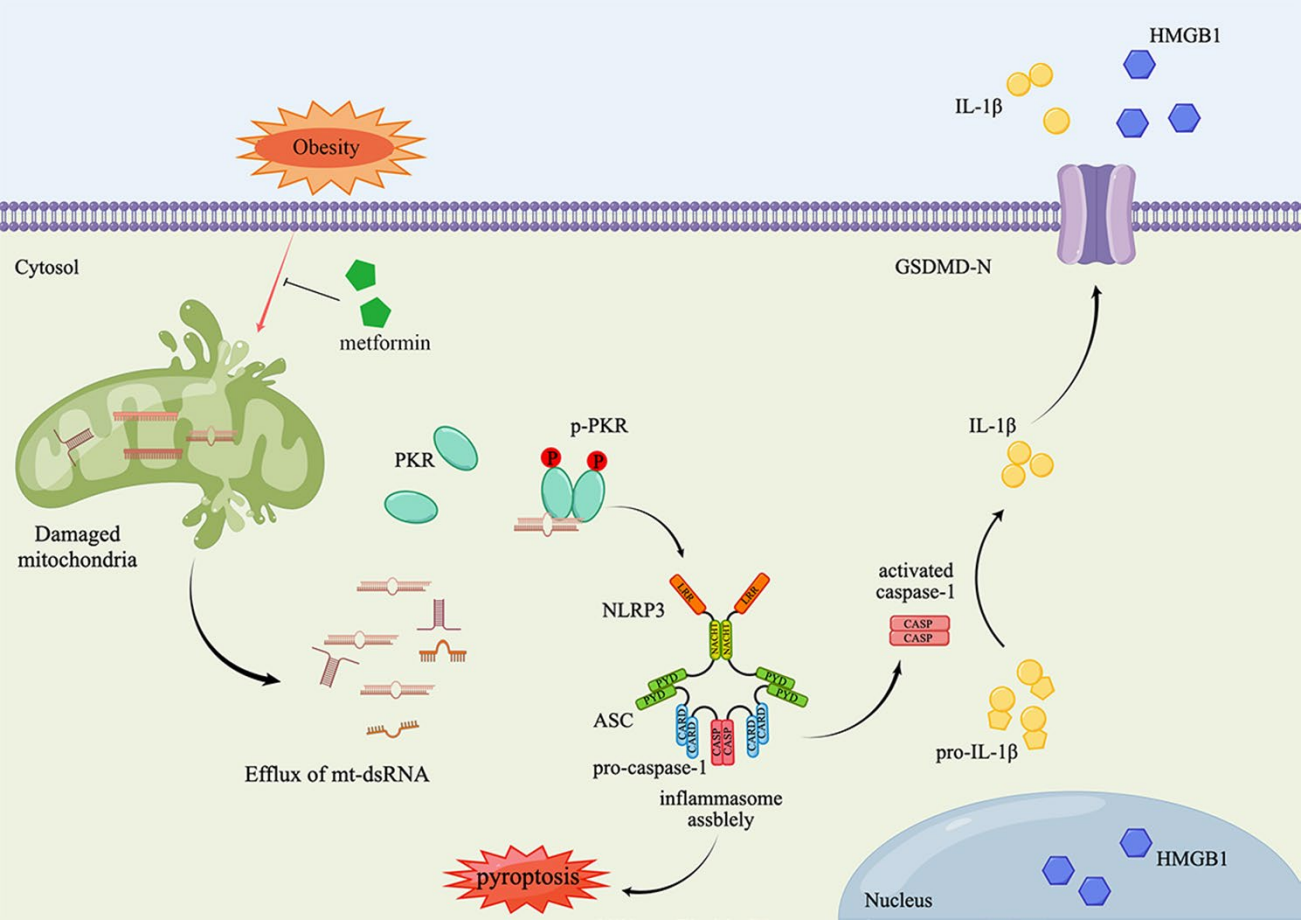
A model for the role of mt-dsRNAs-PKR axis during the metabolic stress in NP cells. Obesity leads to mitochondria damage. The damaged mitochondria release mt-dsRNAs which can activate the PKR resulting in the activation of NLRP3 inflammasome, and NP cell pyroptosis. These harmful effects of obesity can be attenuated by the application of metformin through protecting mitochondrial from hypertriglyceridemia damage. (By Figdraw)

Studies have shown that IVDD is a common concomitant disease in obesity patients [51, 52], and people with obesity at a young age have a greater risk of IVDD than those who develop obesity in middle age [53]. Until now, few studies have focused on the impact of metabolic alterations due to obesity on IVDD and the molecule mechanism between IVDD and obesity remains to be understood. In this study, our results demonstrate that HFD can lead to obesity and to high serum levels of fatty acid, including triglyceride. And the fatty acid leads to mitochondria damage, which increases the content of mt-dsRNAs in the cytoplasm and these mt-dsRNAs act as endogenous activator of PKR to promote NP cell NLRP3 inflammasome activation and IVDD. PKR as a key factor in the activation of NLRP3 inflammasome plays an important role in the IVDD process. Moreover, metformin could alleviate IVDD by attenuating mitochondrial damage and may be used clinically to treat patients with IVDD (Fig. 7).

The degenerative changes of IVD include NP cells losing, ECM degradation, inflammatory microenvironment formation and pro-inflammatory factors secretion [54, 55]. Recent bioinformatic studies have further identified aging-related genes such as FPR1 and UCHL1, which are upregulated in degenerated discs and contribute to inflammatory signaling and cellular senescence, reinforcing the role of metabolic and immune dysregulation in IVDD [56]. Tissue engineering approaches using decellularized matrix hydrogels have shown promise in promoting AF repair and ECM synthesis, providing a complementary strategy to pharmacological interventions [57]. Previous research confirmed that metabolic dysregulation of the cells plays a key role in the pathobiology of IVDD and leads to changes in the ECM component [58]. Numerous studies reported that during obesity the high concentrations of pro-inflammatory cytokines, sugars and circulating lipids could lead to IVD cells damaged [24, 59]. Therefore, how the NP cells sense pathogenic or damage associated signals and respond to these signals which lead to cell dysfunction and ECM microenvironment disturbance is the key to studying IVDD. In this study, we found that the activation of the PKR was increased in IVDD mice model, and PKR−/− mice showed better preservation of NP tissue morphology and less expression of inflammasome-dependent cytokines. Together with the evidence that PKR can sense metabolic homeostasis and active inflammation signal by affecting important inflammatory factor expression [31], these findings suggest that PKR plays a critical role in IVDD as an important molecule that integrates obesity and NP cells pyroptosis.

The NLRP3 inflammasome is a multiprotein complex that is associated with supervision of pathogenic motifs, damage signals, and microenvironmental turbulence [60]. Recent studies have shown that abnormal activation of NLRP3 in response to pathogenic or injury-related stress can promote inflammation of IVD to accelerate the progression of IVDD [40, 61]. The type of PRP used may also influence outcomes; recent evidence suggests that leukocyte-poor PRP (Lp-PRP) may be more effective than leukocyte-rich PRP (Lr-PRP) due to its lower inflammatory cytokine content [62]. In this study, we found that PKR and NLRP3 could interact with each other, which is enhanced during IVDD and leads to abnormal activation of NLRP3 inflammasome and ultimately mediates pyroptosis of NP cells. Considering the important role of NLRP3 inflammasome and HMGB1 in IVDD [40, 63], the discovery of PKR as a key factor in the activation of NLRP3 inflammasome in IVDD has important implications for understanding the pathogenesis of IVDD.

One of the rest significant questions in regard to the relationship between IVDD and obesity is how the PKR response to the hypertriglyceridemia and links the obesity to NP cell pyroptosis since TLR4 is not indispensable for PKR activation. Although PKR was initially shown to act as an intracellular sensor for viral dsRNAs, more recently PKR has been shown to have a broader role as a sensor of danger signal that can be activated by cellular and metabolic stress [31, 64]. Our study suggests that mt-dsRNAs as an endogenous damage signal of obesity are released from damaged mitochondria in NP cells, and these mt-dsRNAs interact with PKR, then lead to PKR phosphorylation level increasing. Moreover, we found that reducing mt-dsRNAs expression simulated the effects of PKR inhibition on NP cell pyroptosis, indicating that mt-dsRNAs were the major activator of PKR during IVDD. Many studies have confirmed that nutrient metabolic disorders such as obesity, type 2 diabetes and metabolic syndrome is co-occurrence of mitochondrial dysfunction [65–67]. Previous studies have shown that mitochondrial damage is associated with various degenerative diseases, including IVDD and osteoarthritis [68, 69]. However, the molecular mechanism of how these damaged mitochondria affect IVDD remains unknown. To the best of our knowledge, this is the first study to demonstrate that mt-dsRNAs can be released from damaged mitochondria during IVDD, and they act as endogenous dsRNAs to facilitate PKR activation and catabolic degenerative changes of NP cells.

Metformin, which is widely used to treat type 2 diabetes, has recently been found to possess anti-inflammatory properties [48]. In vitro studies, we observed that metformin attenuates mitochondria damage and decreases mt-dsRNAs content in the cytoplasm. In the meantime, we also observed inhibition of PA-induced PKR activation and inflammatory cytokine production in metformin-treated NP cells. These findings were further confirmed in vivo experiments, which presented a long-term effect of metformin on the mitochondria protection. Our results indicated that metformin may represent a new therapeutic drug or the prevention and treatment of degenerative disc disease. However, the precise mechanism of its mitochondrial protection effect remains to be clarified.

In summary, this research emphasizes the vital involvement of the mt-dsRNA-PKR axis in NLRP3 inflammasome-mediated NP cell pyroptosis during obesity induced IVDD. Specifically, we observed that PA induced mitochondria damage and mt-dsRNAs efflux into the cytosol, which facilitated the activation of PKR. Moreover, the activated PKR directly interacts with NLRP3 and result in NLRP3 inflammasome activation and NP cell degeneration. In addition, we also found metformin alleviated IVDD progression by attenuating mitochondria damage. Thus, the mt-dsRNAs-PKR axis could be a potential target to improve the therapeutic effect of IVDD and LBP.

### Limitations of study

Our research has several limitations. First, this research demonstrated that metformin could be applied to inhibit IVDD development and progression. However, one important question that we were unable to address in this research is whether metformin is able to delay IVDD progression when IVDD is fully developed. Our long term goal is to conduct a longitudinal study to investigate the effect of metformin on the progression of IVDD when it is fully developed. Another limitation is that we have used healthy and male mice for IVDD studies in the present research, to further find out the mitochondrial protective effects of metformin, we will test the effects of metformin on female mice, on ageing associated IVDD models mice. The last limitation is that other studies have reported that diabetes or high glucose can also cause IVDD. However, the serum glucose level of our obesity mice was also higher than the normal group, which could not be excluded in our study.

## Supplementary Information

Below is the link to the electronic supplementary material.

Supplementary Material 1

Supplementary Material 2

Supplementary Material 3

Supplementary Material 4

## Data Availability

The data generated in the present study may be requested from the corresponding author.
