# Supplementary material for: Obesity-induced NLRP3 inflammasome activation in nucleus pulposus cells accelerates intervertebral disk degeneration
Source: J Orthop Surg Res. 2025 Oct 29;20:934. doi: 10.1186/s13018-025-06382-y (PMC12573823; doi:10.1186/s13018-025-06382-y)
Supplement: Supplementary file 1 — Supplementary Material 1 [file 13018_2025_6382_MOESM1_ESM.docx]

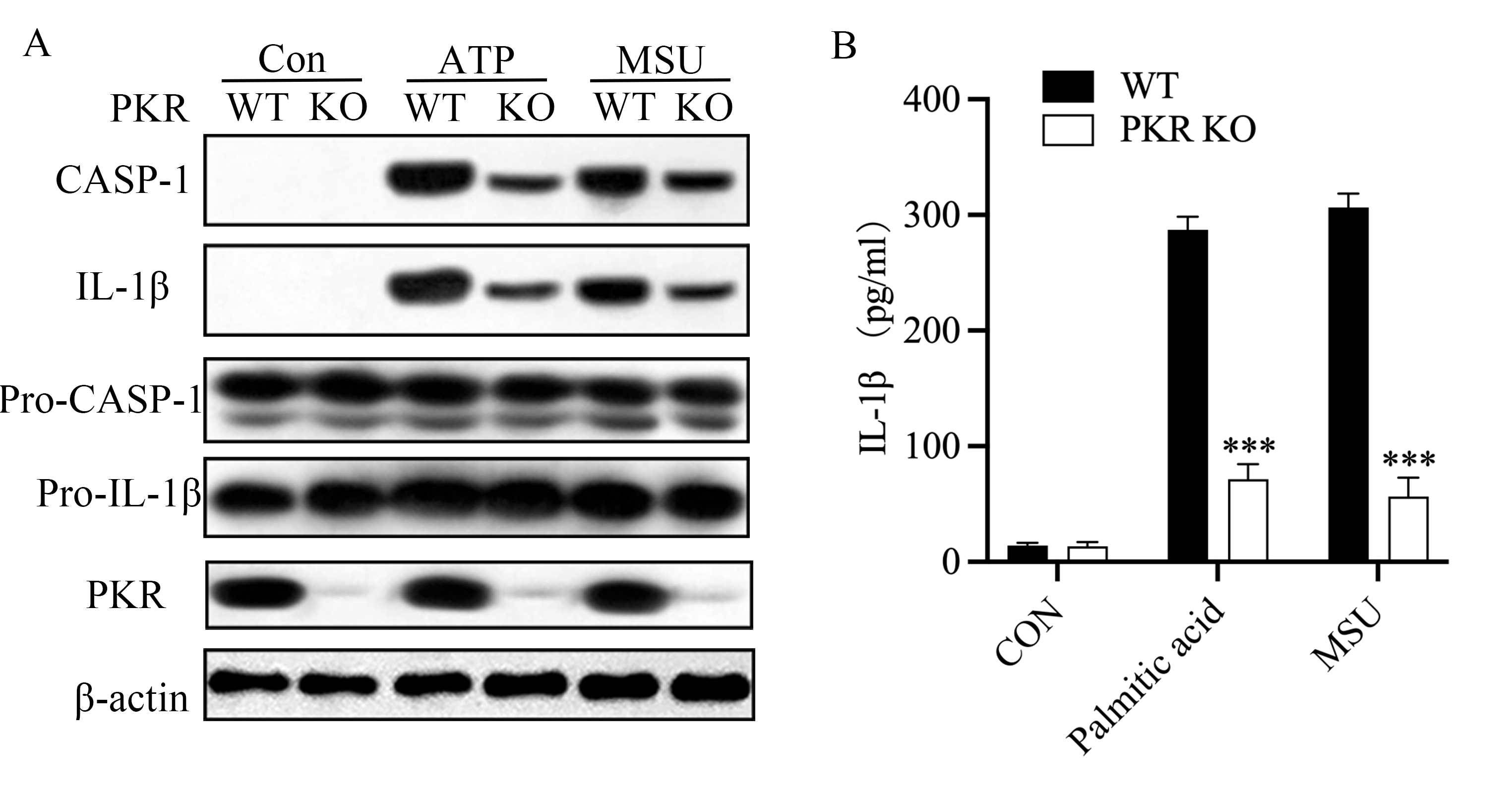


**Supplementary Figure 1. PKR is important for inflammasome activation.**

(A) PKR+/+ or PKR-/- NP cells were stimulated as indicated. Caspase-1 activation and IL-1β cleavage were assessed by Western blot, n=3. (B) PKR+/+ or PKR-/- NP cells were treated with palmitic acid or MSU as indicated. IL-1β levels were measured in the supernatant by ELISA, ***p<0.001 by Student’s t test, n=3.


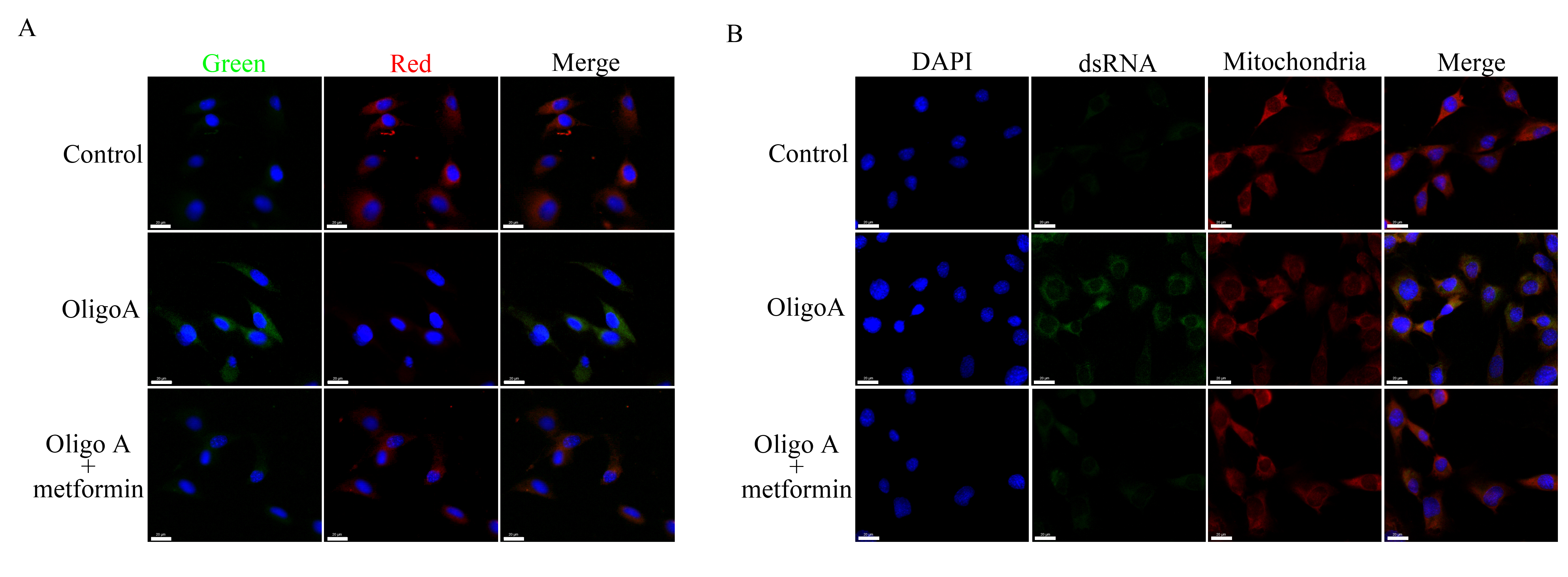


**Supplementary Figure 2. Metformin attenuates mitochondria damage.**

(A) The effect of metformin on mitochondrial membrane potential in OligoA treated NP cells, scale bar=20um, n=3. (B) The effect of metformin on dsRNA expression in OligoA treated NP cells. Mitochondria and nuclei are stained with MitoTracker Deep Red and Hoechst, respectively, scale bars=20um, n=3.
