# Supplementary material for: Obesity-induced NLRP3 inflammasome activation in nucleus pulposus cells accelerates intervertebral disk degeneration
Source: J Orthop Surg Res. 2025 Oct 29;20:934. doi: 10.1186/s13018-025-06382-y (PMC12573823; doi:10.1186/s13018-025-06382-y)
Supplement: Supplementary file 2 — Supplementary Material 2 [file 13018_2025_6382_MOESM2_ESM.docx]

Table S1. Primer sequences for RT-qPCR

| Gene | Forward Primer (5′-3′) | Reverse Primer (5′-3′) |
| --- | --- | --- |
| ND1 | TCCGAGCATCTTATCCACGC | GTATGGTGGTACTCCCGCTG |
| ND4 | TAATCGCACATGGCCTCACA | CATTTGAAGTCCTCGGGCCA |
| ND5 | CAGCACAATTTGGCCTCCAC | TAGTCGTGAGGGGGTGGAAT |
| ND6 | CCCGCAAACAAAGATCACCC | TCTTGATGGTTTGGGAGATTGGT |
| CYTB | TGCATACGCCATTCTACGCT | AGGCTTCGTTGCTTTGAGGT |
| β-actin | GTACTCTGTGTGGATCGGTGG | AACGCAGCTCAGTAACAGTCC |
