# Supplementary material for: Obesity-induced NLRP3 inflammasome activation in nucleus pulposus cells accelerates intervertebral disk degeneration
Source: J Orthop Surg Res. 2025 Oct 29;20:934. doi: 10.1186/s13018-025-06382-y (PMC12573823; doi:10.1186/s13018-025-06382-y)
Supplement: Supplementary file 3 — Supplementary Material 3 [file 13018_2025_6382_MOESM3_ESM.docx]

Table S2. Primer sequences for strand-specific qPCR

| Gene | **Forward Primer (5′-3′)** | **Reverse Primer (5′-3′)** |
| --- | --- | --- |
| ND1 heavy | TCAAACTCAAACTACGCCCTG | CGCAAATGGGCGGTAGGCGTG |
| ND1 light | GTTGTGATAAGGGTGGAGAGG | CGCAAATGGGCGGTAGGCGTG |
| ND4 heavy | CTCACACTCATTCTCAACCCC | CGCAAATGGGCGGTAGGCGTG |
| ND4 light | TGTTTGTCGTAGGCAGATGG | CGCAAATGGGCGGTAGGCGTG |
| ND5 heavy | CTAGGCCTTCTTACGAGCC | CGCAAATGGGCGGTAGGCGTG |
| ND5 light | TAGGGAGAGCTGGGTTGTTT | CGCAAATGGGCGGTAGGCGTG |
| ND6 heavy | TCATACTCTTTCACCCACAGC | CGCAAATGGGCGGTAGGCGTG |
| ND6 light | TGCTGTGGGTGAAAGAGTATG | CGCAAATGGGCGGTAGGCGTG |
| CYTB heavy | CAATTATACCCTAGCCAACCCC | CGCAAATGGGCGGTAGGCGTG |
| CYTB light | GGATAGTAATAGGGCAAGGACG | CGCAAATGGGCGGTAGGCGTG |
| β-actin | ACACAGTGCTGTCTCGTGGTA | CGCAAATGGGCGGTAGGCGTG |
