# Supplementary material for: Obesity-induced NLRP3 inflammasome activation in nucleus pulposus cells accelerates intervertebral disk degeneration
Source: J Orthop Surg Res. 2025 Oct 29;20:934. doi: 10.1186/s13018-025-06382-y (PMC12573823; doi:10.1186/s13018-025-06382-y)

Figure1C

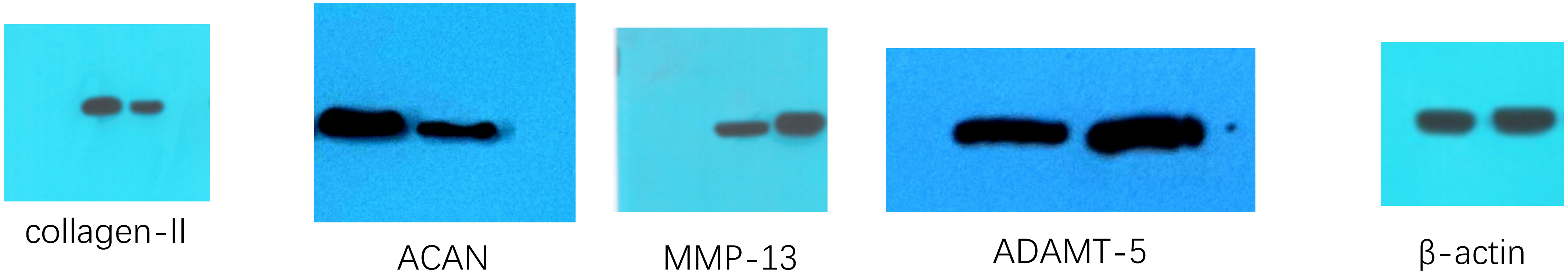

Figure1F

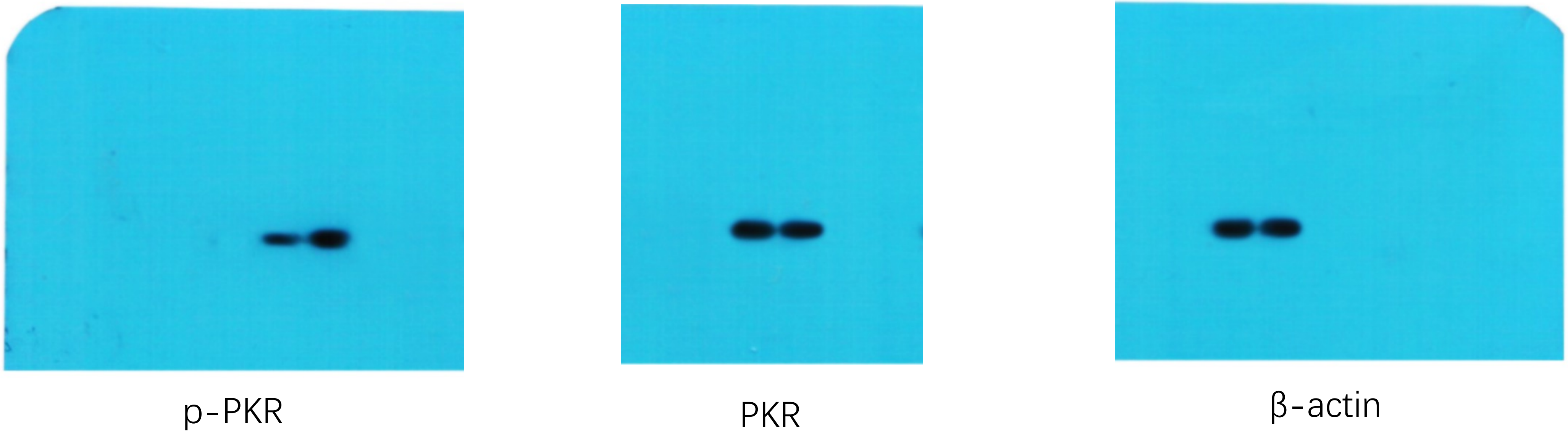

Figure2C

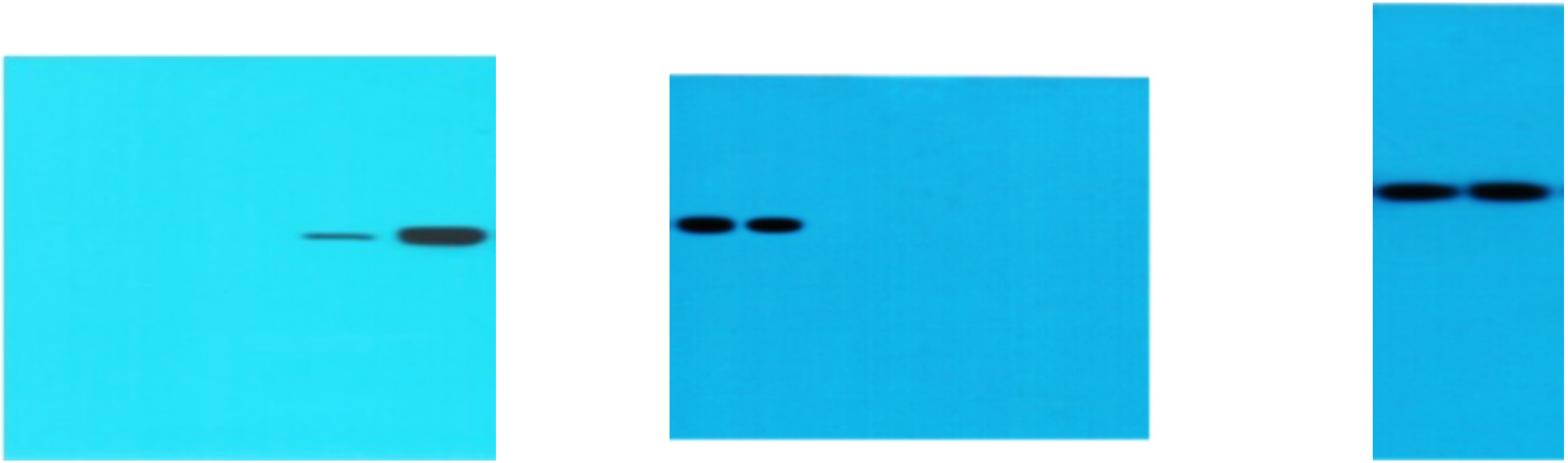

p-PKR PKR β-actin

Figure2F

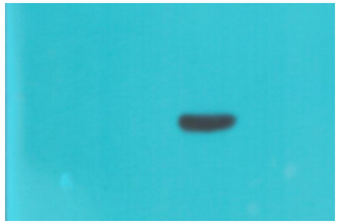

CASP-1

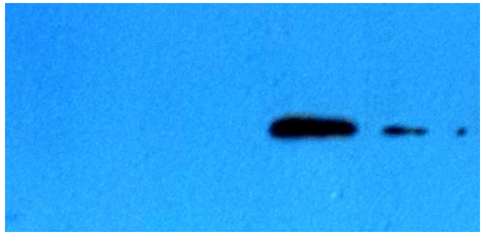

IL-1B

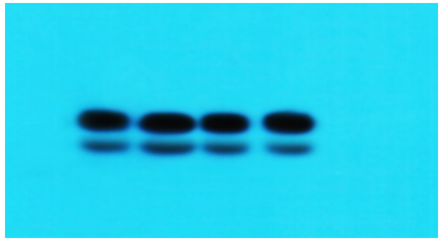

Pro-CASP-1

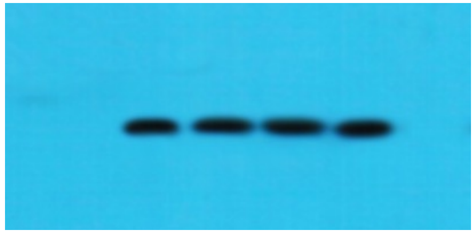

Pro-IL-1B

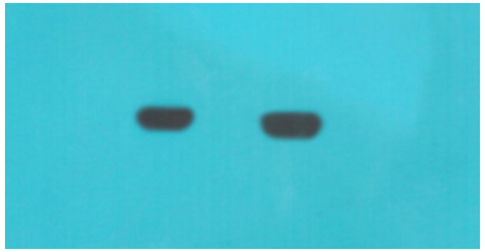

PKR

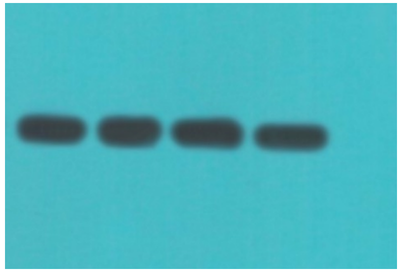

B-actin

Figure3A

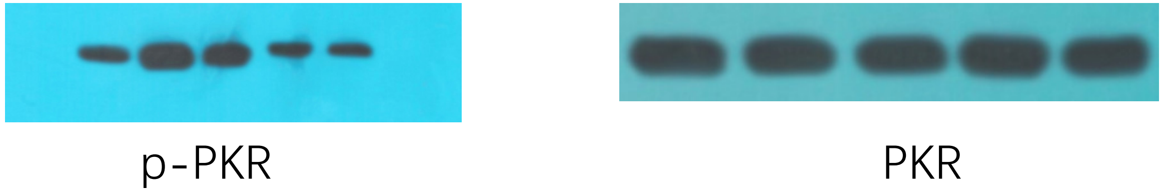

Figure3B

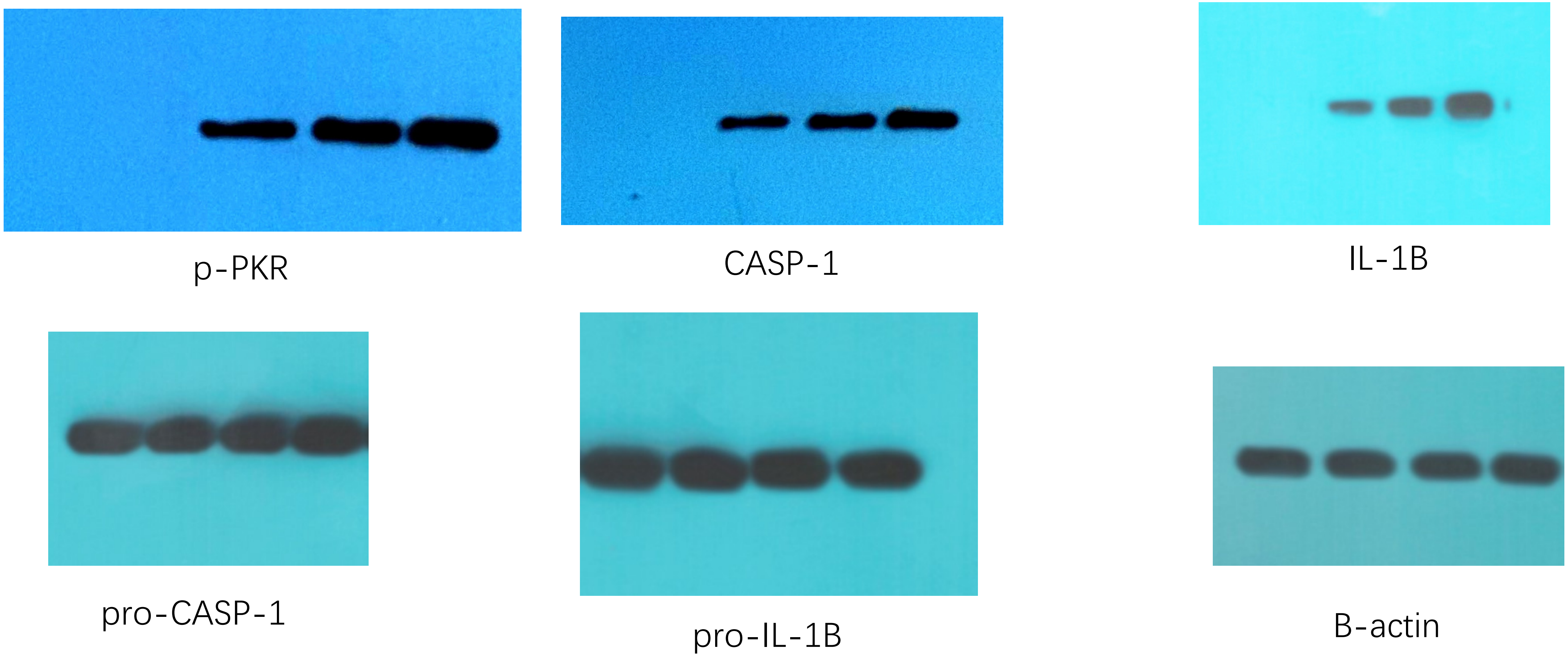

Figure3C

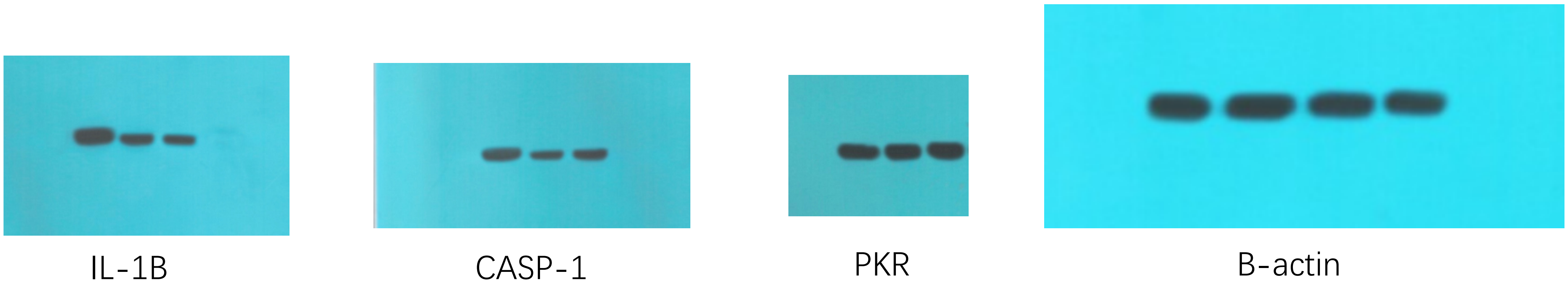

Figure3D

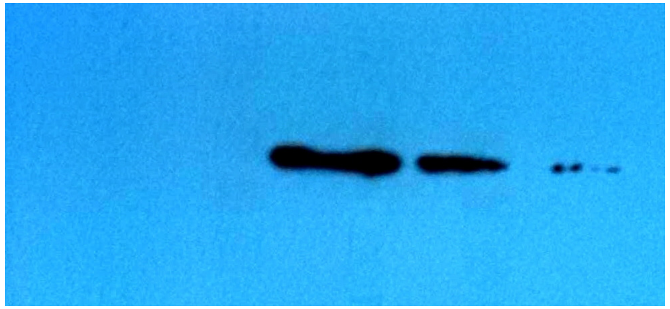

CASP-1

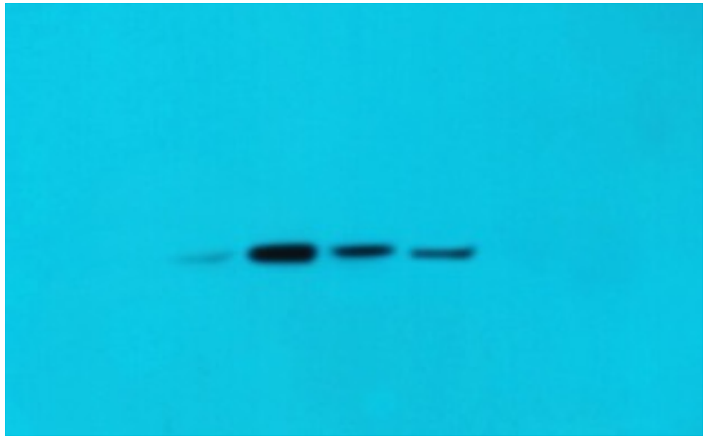

IL-1B

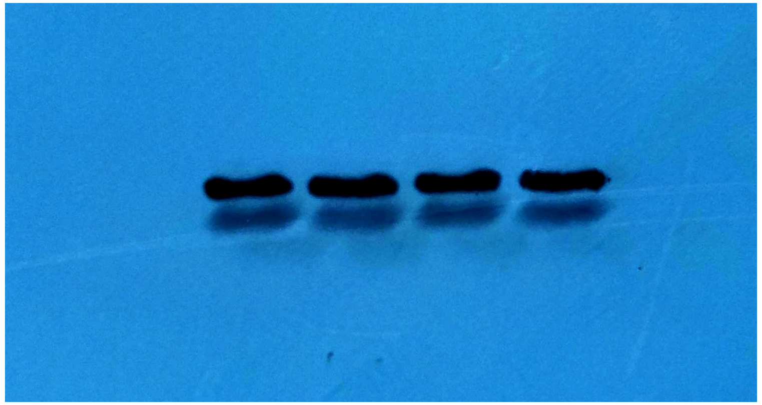

Pro-CASP-1

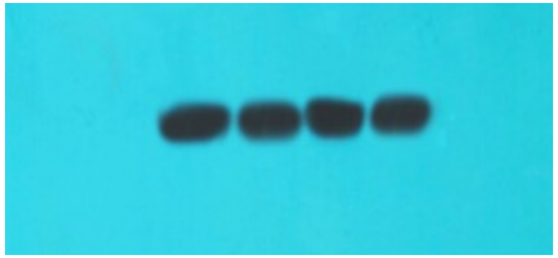

Pro-IL-1B

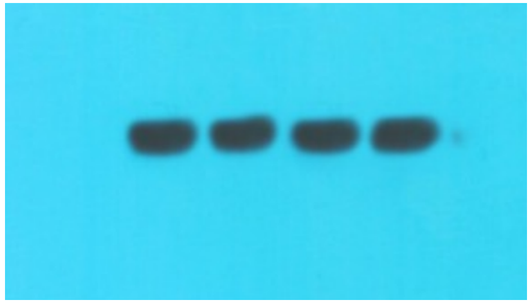

B-actin

Figure3F

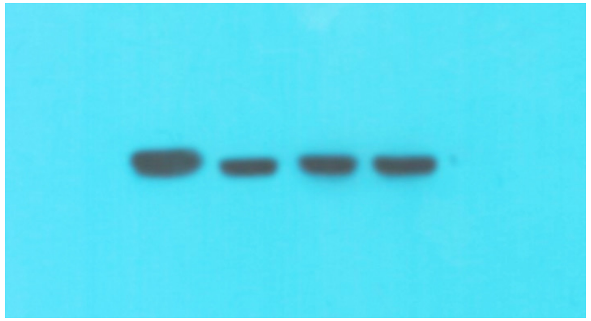

CASP-1

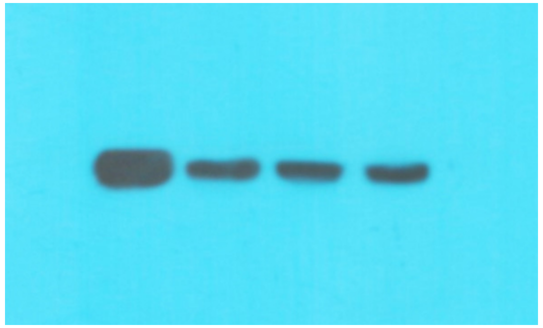

IL-1B

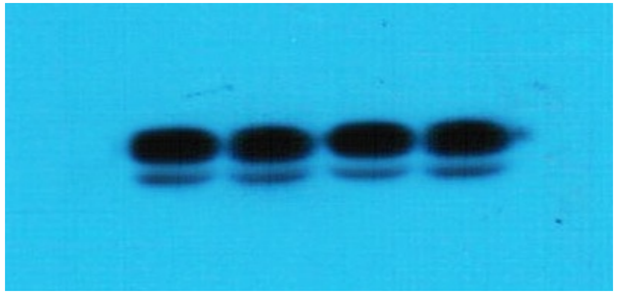

Pro-CASP-1

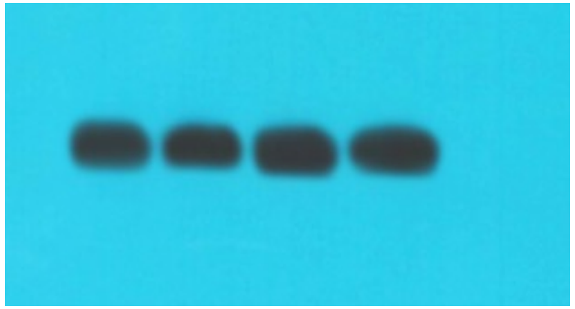

Pro-IL-1

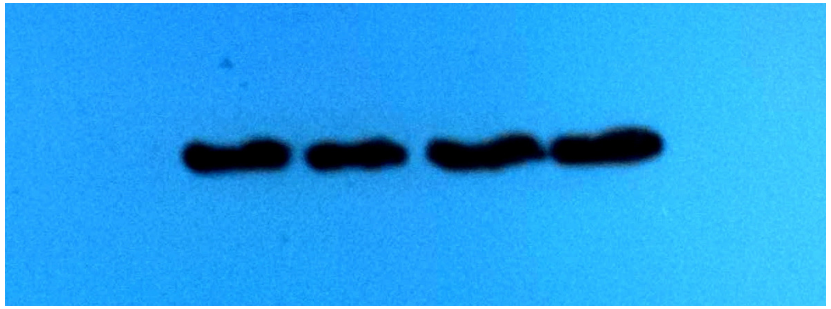

B-actin

Figure3G

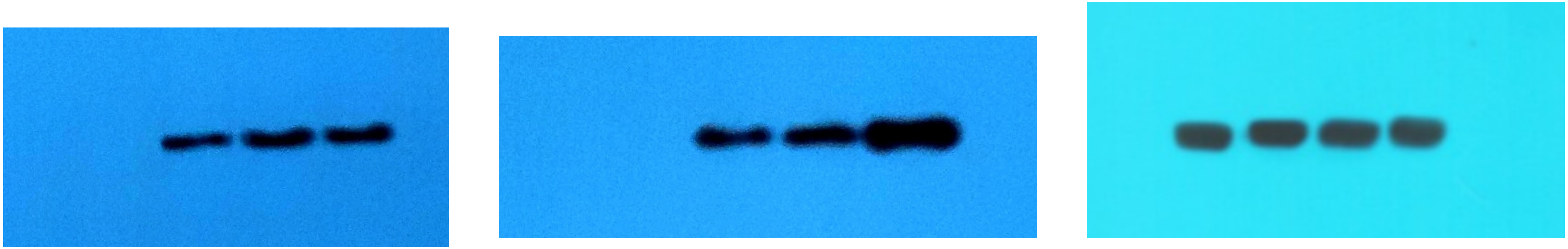

CASP-1 IL-1B B-actin

Figure3H

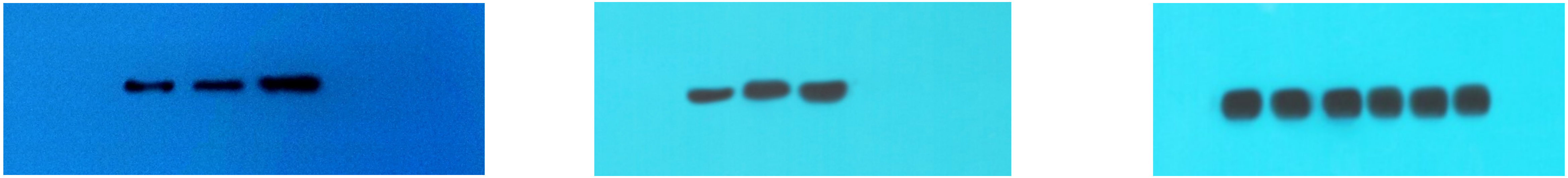

CASP-1 IL-1B Pro-CASP-1

Figure 3I

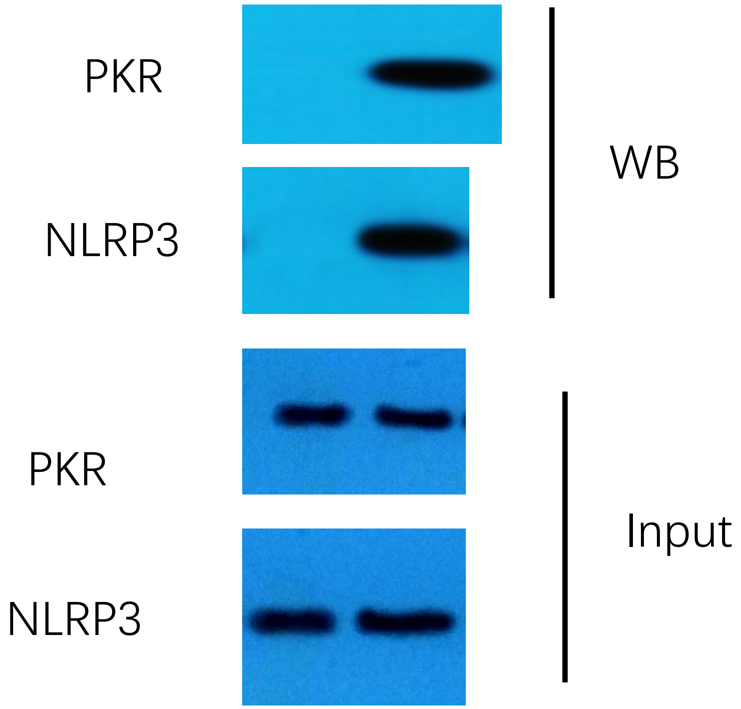

Figure 3J

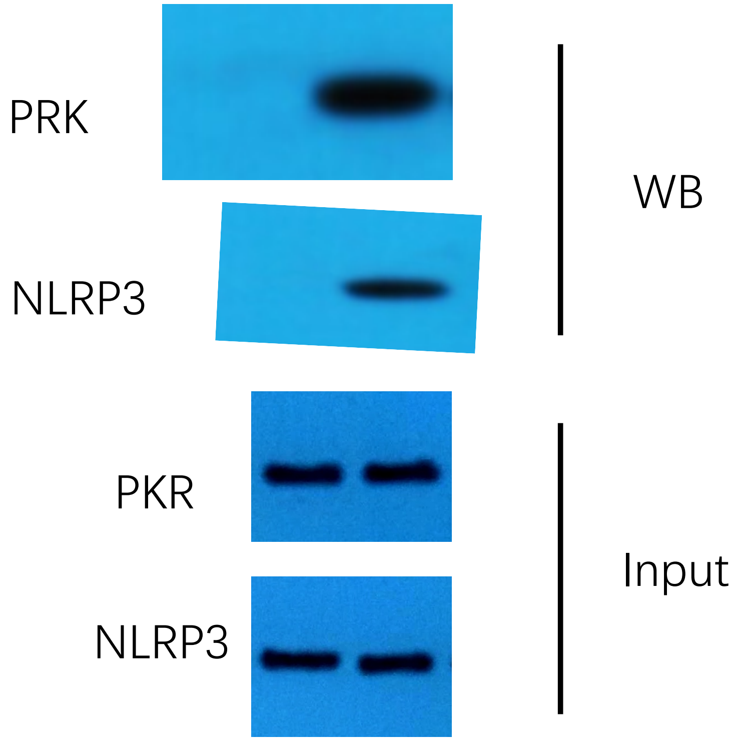

Figure3K

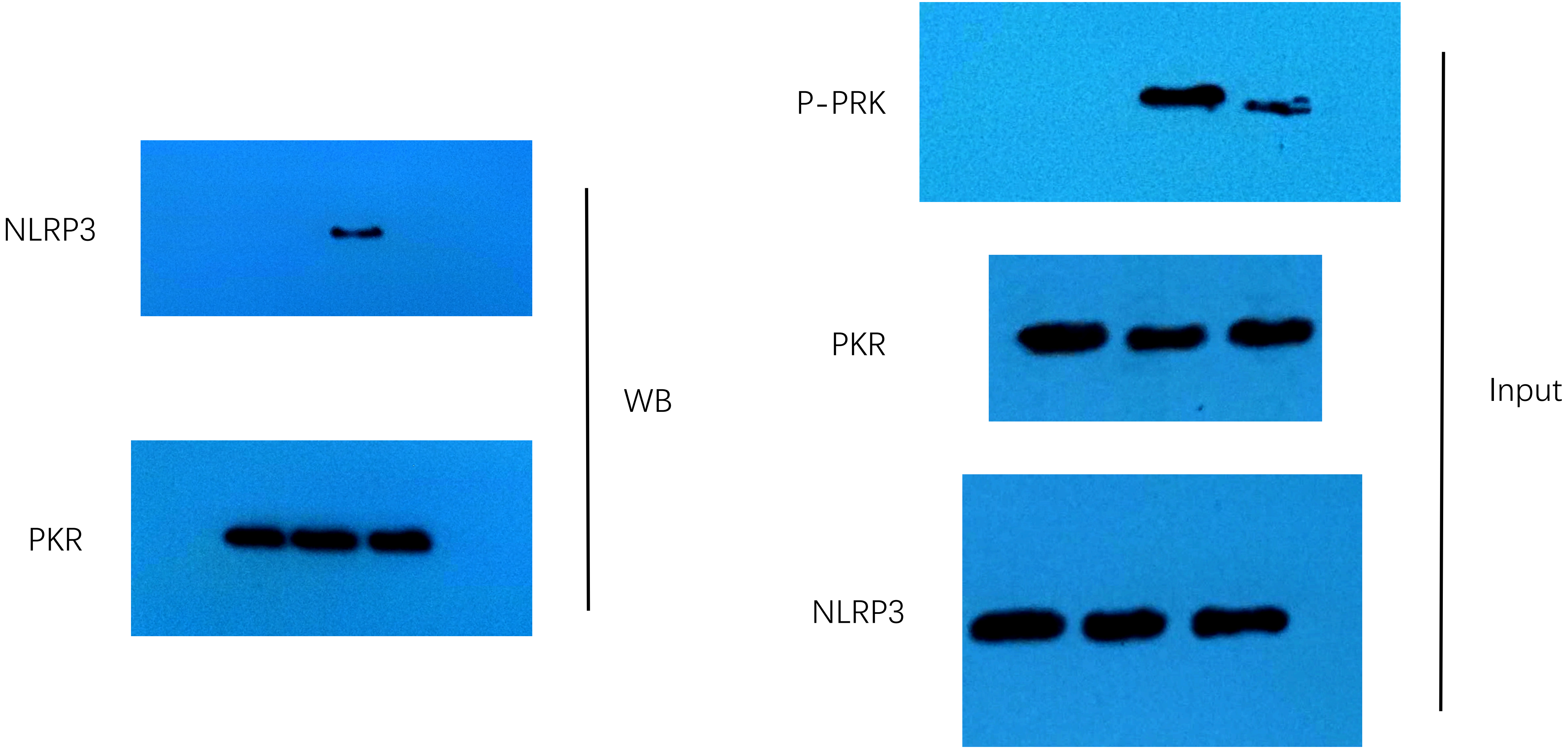

Figure4A

P-PKR

PKR

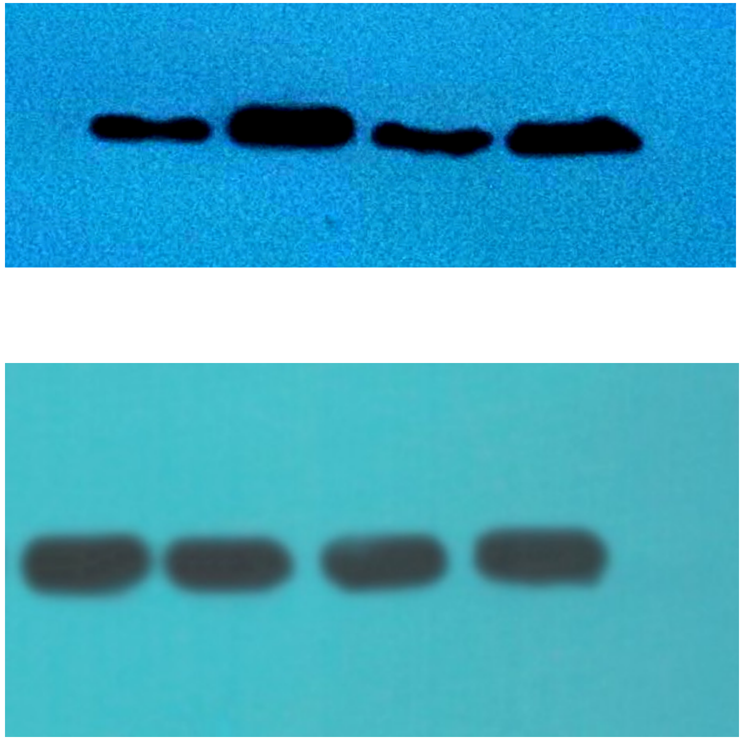

Figure4B

P-PKR

PKR

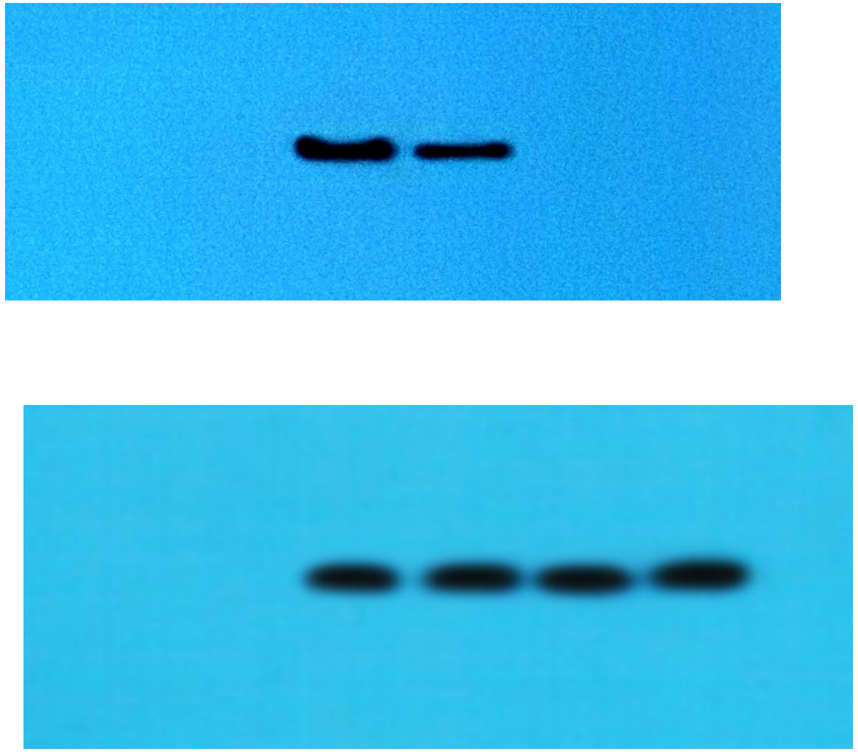

Figure5H

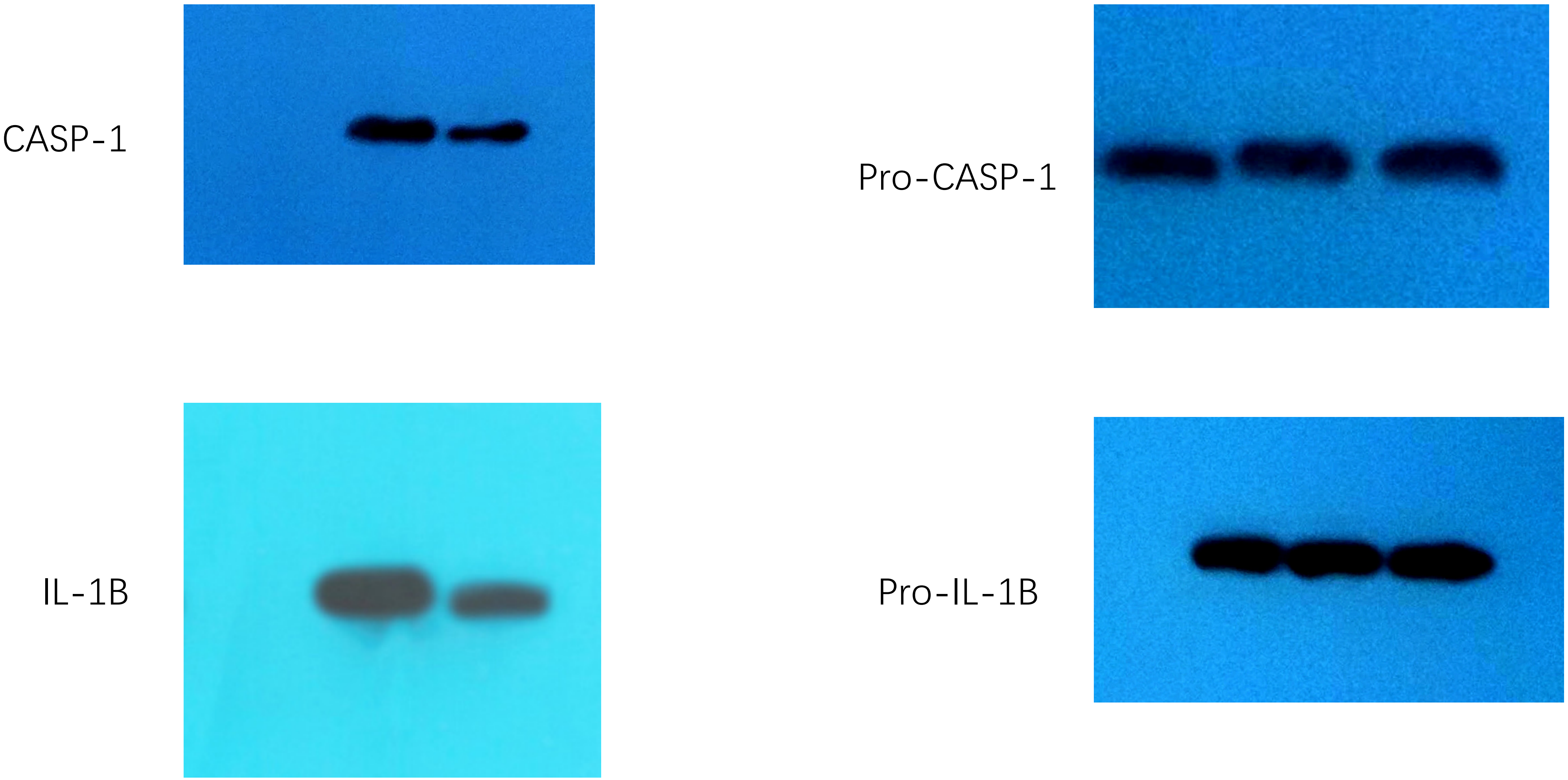

Supplementary figure S1A

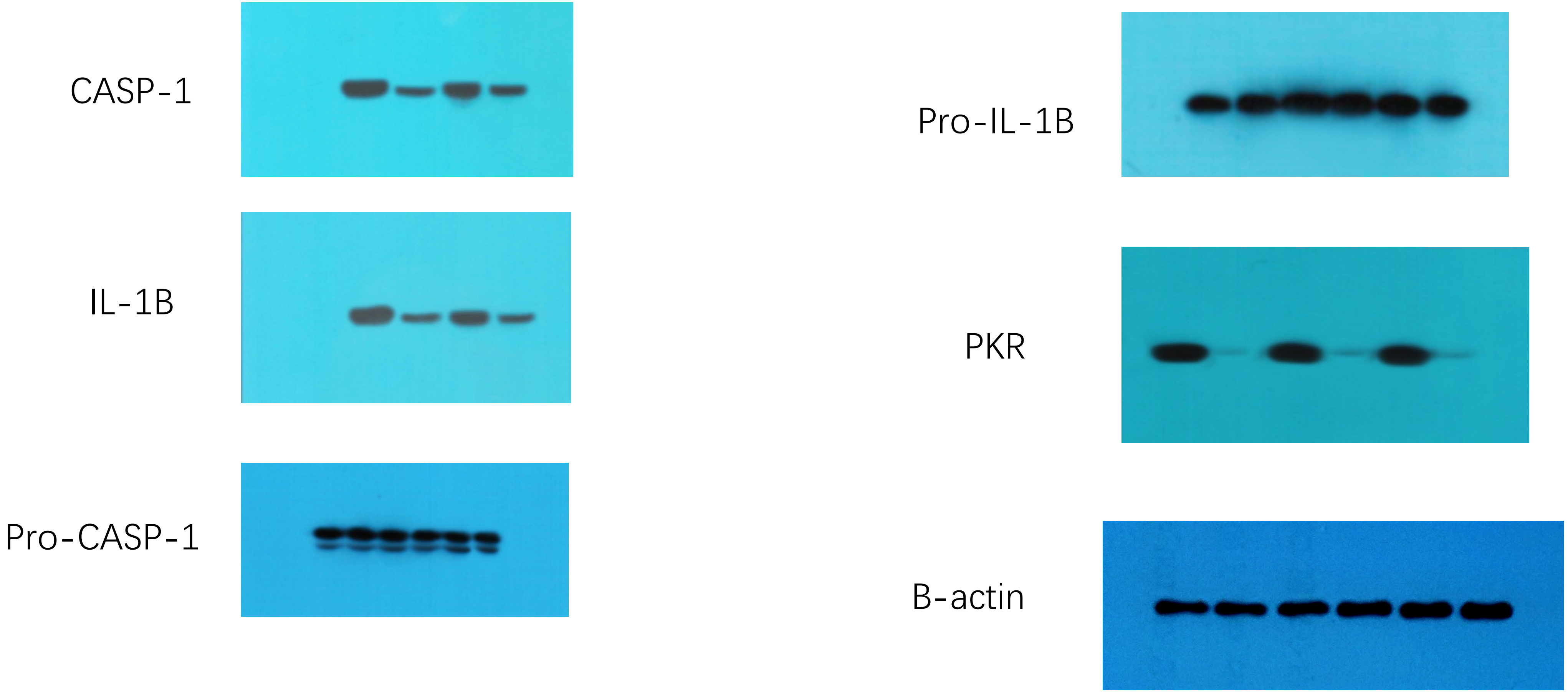

Supplement: Supplementary file 4 — Supplementary Material 4 [file 13018_2025_6382_MOESM4_ESM.pdf]
